# Female hormones prevent sepsis-induced cardiac dysfunction: an experimental randomized study

**DOI:** 10.1038/s41598-022-08889-4

**Published:** 2022-03-23

**Authors:** Alexandre Xerri, Frédéric Gallardo, Frank Kober, Calypso Mathieu, Natacha Fourny, Thi Thom Tran, Jean-Louis Mege, Mervyn Singer, Nathalie Lalevée, Monique Bernard, Marc Leone

**Affiliations:** 1 Aix-Marseille Univ, Service d’anesthésie et de réanimation, Hôpital Nord, Assistance Publique Hôpitaux de Marseille, Chemin des Bourrely, 13015 Marseille, France; 2 Aix-Marseille Univ, CNRS, CRMBM, Marseille, France; 3 Aix-Marseille Univ, INSERM, TAGC, UMR S1090, Marseille, France; 4 CNRS, Marseille, France; 5 Aix-Marseille Univ, Laboratoire d’Immunologie, Hôpital de la Conception, Assistance Publique Hôpitaux de Marseille, 147 boulevard Baille, 13385 Marseille, France; 6 University College London, 4919, Bloomsbury Institute of Intensive Care Medicine, London, UK

**Keywords:** Infection, Infectious diseases, Experimental models of disease, Cardiovascular biology

## Abstract

Although epidemiologic research has demonstrated significant differences in incidence and outcomes of sepsis according to sex, their underlying biological mechanisms are poorly understood. Here, we studied the influence of hormonal status by comparing in vivo cardiac performances measured by MRI in non-ovariectomized and ovariectomized septic female rats. Control and ovariectomized rats were randomly allocated to the following groups: sham, sepsis and sepsis plus landiolol. Sepsis was induced by caecum ligation and punction (CLP). Landiolol, a short-acting selective β1-adrenergic blocker improving the in vivo cardiac performance of septic male rats was perfused continuously after sepsis induction. Cardiac MRI was carried out 18 h after induction of sepsis to assess in vivo cardiac function. Capillary permeability was evaluated by Evans Blue administration and measurement of its tissue extravasation. Variation in myocardial gene and protein expression was also assessed by qPCR and western-blot in the left ventricular tissue. Sepsis reduced indexed stroke volume, cardiac index and indexed end-diastolic volume compared to sham group in ovariectomized females whereas it had no effect in control females. This was associated with an overexpression of JAK2 expression and STAT3 phosphorylation on Ser727 site, and an inhibition of the adrenergic pathways in OVR females. Landiolol increased the indexed stroke volume by reversing the indexed end-diastolic volume reduction after sepsis in ovariectomized females, while it decreased indexed stroke volume and cardiac index in control. This was supported by an overexpression of genes involved in calcium influx in OVR females while an inactivation of the β-adrenergic and a calcium efflux pathway was observed in control females. Sepsis decreased in vivo cardiac performances in ovariectomized females but not in control females, presumably associated with a more pronounced inflammation, inhibition of the adrenergic pathway and calcium efflux defects. Administration of landiolol prevents this cardiac dysfunction in ovariectomized females with a probable activation of calcium influx, while it has deleterious effects in control females in which calcium efflux pathways were down-regulated.

## Introduction

Epidemiological studies have shown that both incidence and outcome of sepsis differ according to sex, with a protective effect of female^1–3,5,6^. Interestingly, this difference of outcome was not found in old patients, which suggests an influence of hormonal status^2^.

This influence is exerted in particular at the cardiovascular level. In a murine model of sepsis induced by cecal ligation and punction (CLP), Chen et al. have previously described a sex dimorphism of the septic cardiac dysfunction^7^. Our team showed in vivo that female sex was protective for cardiac dysfunction using a model of rat undergoing CLP^8^.

Hence, there is an overall accumulation of clues suggesting a crucial role of the hormonal status in the control of sepsis-related cardiovascular dysfunction, as it is in other experimental models of cardiovascular dysfunction like trauma-hemorrhage and myocardial ischemia–reperfusion^9,10^. This prompted us to investigate the effect of ovariectomy on the cardiovascular response to sepsis.

In our previous study, we evaluated the influence of sex on the response to a promising treatment in sepsis, the landiolol, a short-acting selective β1-adrenergic blocker. Administration of landiolol improved the in vivo cardiac performance of septic male rats, whereas deleterious effects were found in female rats^8^.

We thus compared in vivo cardiac performances measured by MRI in control and ovariectomized septic female rats, treated by landiolol. Capillary permeability was also evaluated by Evans Blue administration and measurement of its tissue extravasation. Finally, cardiac gene and protein expression was investigated in the left ventricular tissue.

## Methods

### Animals and surgical procedures

Female Wistar rats (8–9-week-old, Charles River, Saint-Germain sur l’Arbresle, France) were housed for a 5–7-day acclimatization period in a temperature and light controlled room with free access to water and food. All animal procedures were conducted in accordance with the National Guidelines for Care and Use of Laboratory Animals in conformity with the 2010/63 EU directive and with the approval of the Institutional Animal Care Committee of Aix-Marseille University (APAFIS number 17814–2019012215408140). Bilateral ovariectomy procedure is reported in the Supplemental Data. Anaesthetic and CLP procedures were performed as previously described^8^.

### Experimental protocol

**Figure 1:**
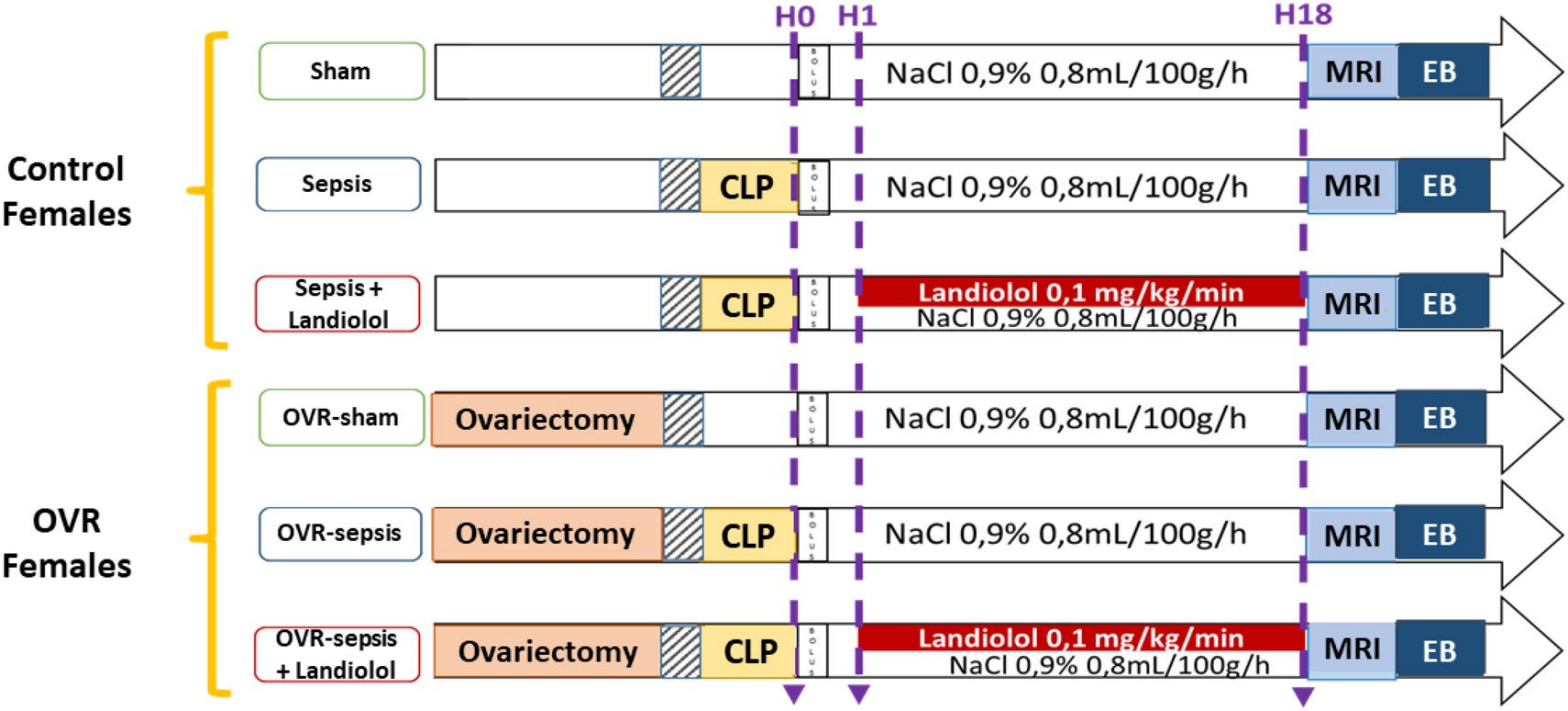
Experimental protocol. *OVR* Bilateral ovariectomy, *CLP* Caecum ligation and punction, *Bolus* NaCl 0.9% 1 mL/100 g/h in 30 min, *MRI* magnetic resonance imaging, *EB* injection of 80 mg/kg of Evans Blue.

Figure 1 summarizes the experimental protocol. Ovariectomized (OVR) and control female rats were randomized to undergo a control laparotomy or the induction of sepsis by caecum ligation and punction (CLP). A delay of 3 to 4 weeks after the ovariectomy was respected. One hour after CLP, they were again randomized to receive landiolol (AOP Orphan, Vienna, Austria) diluted in n-saline and infused at 0.1 mg/kg/min or *n*-saline (0.8 mL/kg/h). The infusion volume was similar in all groups. Six groups were assessed: sham (n = 7), sepsis (n = 8), sepsis plus landiolol (n = 7), OVR-sham (n = 7), OVR-sepsis (n = 6), and OVR-sepsis plus landiolol (n = 7). Eighteen hours after CLP, female rats were anesthetized using 1.8–2% isoflurane. The caudal ventral artery was cannulated (24-gauge catheter) for blood pressure recording (TruWave pressure; Edwards Lifesciences, Irvine, CA). Systolic blood pressure (SBP), diastolic blood pressure (DBP), mean blood pressure (MBP) and heart rate (HR) were recorded at H18. Body weight variations between H0 and H18 were measured. Cardiac MRI was performed 18 h after CLP to assess the in vivo cardiac function (cine-MRI). Immediately after cardiac MRI, 2 mL/kg of 4% Evans Blue (EB) solution were injected to female rats. After 30 min, lungs were removed for subsequent measurement of EB tissue concentration, and hearts were frozen for subsequent quantification of gene and protein expression. The number of rats used for each part of the protocol are detailed in the Supplementary Data (Supplementary Fig. 1).

### MRI assessment of in vivo cardiac function

MRI was performed 18 h after CLP, immediately after arterial cannulation. During MRI, inhalation anaesthesia was maintained with 1.5–2% isoflurane in 1.5 L/min oxygen continuously delivered through a face mask as previously described^8^. Details are provided in the Supplementary Data.

End-diastolic volume (EDV), end-systolic volume (ESV), stroke volume (SV), left ventricle ejection fraction (LVEF), left ventricular mass (LVM), mean wall thicknesses in diastole and systole, and systolic wall thickening (WTn) were calculated from the volume measurements, as previously described^11^. Cardiac output (CO) was calculated as CO = HR × SV. EDV (indexed EDV (EDVi)), ESV (indexed ESV (ESVi)), SV (indexed SV (SVi)), and CI were indexed to weight. Image analyses were performed using a home-made program running in IDL environment.

### In vivo assessment of vascular permeability

Immediately after cardiac MRI, 2 mL/kg of 4% EB solution was injected to female rats, corresponding to a dose of 80 mg/kg of EB. This was calculated so that all injected EB is bound to albumin^12^. Female rats were maintained 30 min under anaesthesia to insure a distribution of EB to the vascular system. At 30 min, female rats were euthanized by 100 mg/kg of phenobarbital. Medial laparotomy was then performed, and the abdominal aorta was severed just below the renal arteries. Twenty millilitres of phosphate buffered saline at 4 °C were injected in the jugular vein to wash the blood and remaining EB from the vascular system. After bilateral thoracotomy cranial pulmonary lobe were removed for analysis.

Removed tissues were incubated 48 h at 55 °C with 4 mL/g of wet tissue of formamide, in order to extract the EB from the tissue. The EB/formamide mixtures were then centrifuged for 15 min at 4000 rpm. Supernatant was collected and absorbance at 610 nm was measured. Extravasated EB concentration per mg tissue was calculated.

### Plasma samples

Blood samples were collected through the jugular vein before CLP and through arterial catheter after cardiac-MRI but before EB injection, centrifuged (2000*g*, 12 min, 4 °C), and the resulting plasma aliquoted and stored at − 80 °C until biochemical analysis.

### RNA extraction and quantitative reverse transcriptase-polymerase chain reaction

Hearts, removed immediately after euthanasia, were dissected, frozen and stored at − 80 °C until analyses. Total RNA was isolated from the left ventricles and qPCR experiments were performed as previously described^13^. Primers used for trancripts detection are listed in Supplementary Table 1.

### Protein extraction and western-blot assay

Lysates were prepared from frozen LVs with Precellys lysing kit (Bertin Technologies) in lysis buffer (1% Triton X-100, 50 mM HEPES, 150 mM NaCl, 25 mM NaF, 1 mM EDTA, 1 mM EGTA, 10 μM ZnCl_2_, 1 mM sodium orthovanadate) containing 4% protease inhibitor cocktail (Sigma-Aldrich). After centrifugation (14,000 rpm, 4 °C, 30 min), lysates were collected and quantified with Q-Bit protein assay (Thermo). Protein extracts were denatured in SDS loading buffer supplemented with DTT. For each sample, 50 µg total protein was separated by NuPAGE^®^ Bis–Tris gels (4–12%) (Invitrogen) electrophoresis and transferred to nitrocellulose membrane using the iBlot2^®^ gel transfer system (Life Technologies). Membranes were blocked in 5% skim milk dissolved in Tris-buffered saline (TBS) then incubated overnight at 4 °C in TBS with 5% BSA with antibodies: Jak2 (3230, 1/500), phosphorylated-Stat3 (Tyr705) (9145, 1/2000) and phosphorylated-Stat3 (Ser727) (9134, 1/500) and actin (4968, 1/1000) (all from Cell Signaling). The blots were washed then incubated with HRP-conjugated secondary antibodies. The protein bands were detected using the enhanced chemiluminescence reagent (ECL Prime Western Blotting System, Cytiva) and visualized using the iBright CL750 Imaging System (Thermo). Signal analysis was performed with ImageJ software (NIH, Bethesda, MD, USA).

### Statistical analyses

Data are expressed as means ± SEM. After statistical evaluation, the number of rats required was 6 per group to show a 30% increase in SV by landiolol with a power of 80% and an alpha risk of 5%. Significant differences between groups were determined using two-way analysis of variance followed by Sidak post hoc testing with GraphPad Prism software (GraphPad Prism 7.03, La Jolla, CA). A *p* value of less than 0.05 was considered statistically significant.

### Animal research

Reporting of In Vivo Experiments:The authors declare that this study is reported in accordance with ARRIVE guidelines.

## Results

### General effect of ovariectomy

OVR females had an increased weight gain compared to control (p < 0.001 for sham groups; p < 0.05 for sepsis groups and p < 0.01 for sepsis plus landiolol groups) (Table 1). Body temperature and hemodynamic variables were similar in all groups.

**Table 1:** Physiological and hemodynamic parameters 18 h after CLP.

Measured parameters	Control			OVR		
	Sham (n = 6)	Sepsis (n = 5)	Sepsis + Landiolol (n = 5)	Sham (n = 6)	Sepsis (n = 6)	Sepsis + Landiolol (n = 6)
Age (weeks)	11.2 ± 0.4	11.6 ± 0.9	11.7 ± 1.2	12.3 ± 1.4	12.2 ± 1	12.2 ± 1.2
Initial weight (g)	183 ± 16	196 ± 20	203 ± 18	191 ± 17	185 ± 26	198 ± 26
∆ weight post-OVR (g)	38 ± 12	42 ± 12	48 ± 29	96 ± 16^†††^	81 ± 11^†^	99 ± 28^††^
∆ weight post-CLP (g)	11 ± 8	16 ± 5	19 ± 8	19 ± 7	20 ± 5	10 ± 4
RR (cycles/min)	50 ± 3	56 ± 8	53 ± 9	55 ± 12	56 ± 12	61 ± 13
Temperature (°C)	35.9 ± 0.4	35.6 ± 0.4	34.5 ± 1.4	35.8 ± 0.4	35.1 ± 0.5	35.5 ± 1.3
SBP (mmHg)	126 ± 6	113 ± 3	92 ± 16**	126 ± 12	94 ± 12*	102 ± 25
DBP (mmHg)	90 ± 9	77 ± 17	59 ± 18	84 ± 9	68 ± 5	69 ± 21
MBP (mmHg)	102 ± 6	89 ± 12	70 ± 17**	98 ± 9	77 ± 7	80 ± 21
HR (bpm/min)	389 ± 37	397 ± 24	358 ± 31	382 ± 25	389 ± 52	357 ± 38

*Age* age at the time of CLP, *∆ weight post-OVR* weight difference between OVR and CLP procedures, *∆ weight post-CLP* weight difference between CLP and 18 h after, *RR* respiratory rate, *SBP* systolic blood pressure, *DBP* diastolic blood pressure, *MBP* mean blood pressure, *HR* heart rate. Data are expressed as means ± SD.

^†^p < 0.05; ^††^p < 0.01; ^†††^p < 0.001 vs control of corresponding group.

*p < 0.05; **p < 0.01 vs control sham and vs OVR-sham, respectively.

**Figure 2:**
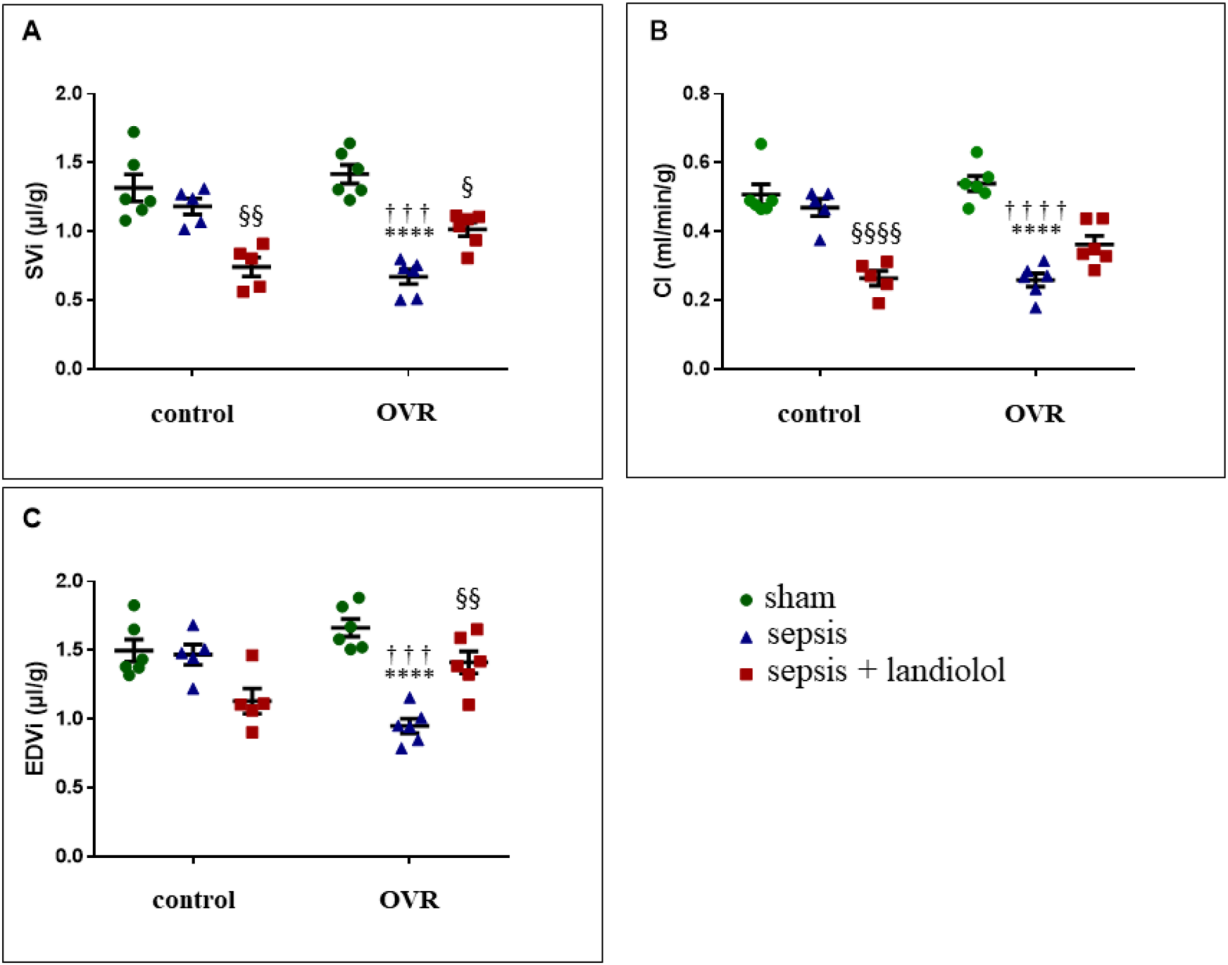
Left ventricular cardiac parameters assessed by MRI of sham (n = 6), sepsis (n = 5), sepsis plus landiolol (n = 5), OVR-sham (n = 6), OVR-sepsis (n = 6) and OVR-sepsis plus landiolol (n = 6). (**A**) Indexed stroke volume (SVi). (**B**) Cardiac index (CI). (**C**) Indexed end-diastolic volume (EDVi). Data are expressed as means ± SEM. ****p < 0.0001 *vs* sham and *vs* OVR-sham, respectively. ^§^p < 0.05; ^§§^p < 0.01; ^§§§§^p < 0.0001 *vs* sepsis and OVR-sepsis, respectively. ^†††^p < 0.001; ^††††^p < 0.0001 *vs* control of corresponding group.

After weight indexing, cardiac variables assessed by MRI were unchanged between OVR and control females (Fig. 2). There were no differences of left ventricular mass between OVR and control females of corresponding groups.

No significant differences were found in EB concentrations in lung tissue between OVR and control females of corresponding groups. There were no differences in lactate and procalcitonin plasma concentrations as well (Table 2).

**Table 2:** Biological parameters 18 h after CLP.

Biological parameter	Control			OVR		
	Sham (n = 5)	Sepsis (n = 5)	Sepsis + Landiolol (n = 4)	Sham (n = 6)	Sepsis (n = 6)	Sepsis + Landiolol (n = 5)
Evans Blue (ng/mg tissue)	59 ± 41	92 ± 22	108 ± 61	37 ± 28	125 ± 67	90 ± 50
Lactate (ng/µL)	74 ± 35	145 ± 66	111 ± 70	79 ± 44	191 ± 83	173 ± 92
Procalcitonin (pg/µL)	14 ± 10	20 ± 12	41 ± 26	20 ± 22	48 ± 38	39 ± 24

*Evans Blue* Evans Blue tissue concentration in pulmonary right cranial lobe, *Lactate* lactate plasma concentrations, *Procalcitonin* procalcitonin plasma concentration. Data are expressed as means ± SD.

### Effects of sepsis on cardiac function in OVR females

Sepsis did not affect the weigh, respiratory rate and body temperature at H18. In control females, hemodynamic variables were similar after the sepsis procedure. In OVR females, sepsis decreased systolic blood pressure (p < 0.05), as compared with sham (Table 1).

Sepsis did not affect the cardiac performances in control females; no differences in cardiac index (CI), indexed stroke volume (SVi), and indexed end diastolic volume (EDVi) were reported, as compared with sham. In contrast, in OVR females, sepsis decreased the CI by 52% (p < 0.0001) and SVi by 53% (p < 0.0001), reflecting the global performance of the heart.

This change did not affect the cardiac systolic function, as there was no significant difference in left ventricular ejection fraction (LVEF), but the diastolic function: EDVi was decreased by 43% (p < 0.0001), as compared with sham (Fig. 2). Of note, sepsis did not affect left ventricular mass in both control and OVR females.

In control females, sepsis did not increase EB tissue concentration, as compared with sham. Similarly, in OVR females, sepsis did not increase EB concentration in lung tissues, as compared with sham (p = 0.054). No significant differences were found in lactate and procalcitonin plasma concentrations after sepsis between control and OVR females (Table 2).

In order to identify transcriptional changes associated with ovariectomy in sepsis-induced cardiac dysfunction, we analysed by qPCR the expression in the left ventricle of genes encoding signalling proteins known for their role in cardiac and immune functions. In both control and OVR females, sepsis increased the myocardial expression of genes encoding protein associated to inflammation, interleukin-6 (IL-6), IL-10 and IL-18, to cell cycle and survival, Janus Kinase 2 (JAK2) and signal transducer and activator of transcription 3 (STAT3) (Table 3 and Supplementary Table 2). Regarding pro-apoptotic genes, sepsis increased mitogen activated protein kinase 14 (MAPK14) in OVR females and tumor necrosis factor- α (TNF-α) in control females (Table 3 and Supplementary Table 2).

**Table 3:** Gene expression variation after sepsis.

Biological processes	Gene	Effect of sepsis		Effect of landiolol in sepsis		Effect of sepsis + landiolol	
		Control	OVR	Control	OVR	Control	OVR
Pro-inflammatory molecules	JAK2	**↗**	**↗**	=	=	**↗**	**↗**
	STAT3	**↗**	**↗**	=	=	**↗**	**↗**
	IL-1b	**↗**	=	=	**↗**	**↗**	**↗**
	IL-6	**↗**	**↗**	=	=	**↗**	**↗**
	IL-18	**↗**	**↗**	=	=	**↗**	**↗**
	TNF-α	**↗**	=	=	=	**↗**	=
Anti-inflammatory molecules	IL-10	**↗**	**↗**	=	=	**↗**	**↗**
Apoptosis	MAPK14	=	**↗**	=	=	=	=
Adrenergic signalling pathway	CREM	**↗**	**↗**	=	**↘**	=	=
	ADRA1A	=	**↘**	=	=	=	=
	PLCB4	=	**↘**	=	=	=	=
	GRK5	=	**↗**	=	=	**↗**	=
	AKAP6	**↘**	**↘**	=	=	**↘**	=
Calcium signalling	SLC8A1	**↘**	=	=	=	=	=
	ATP2B2	=	**↘**	=	Norm	**↘**	=
	RYR2	=	=	=	**↗**	=	=
	RYR3	=	=	=	**↗**	=	=
	SERCA2	=	=	=	=	**↘**	=
	SERCA3	**↘**	**↘**	Norm	=	=	=
	CACNA1C	=	=	=	**↗**	=	=
	PLN	=	=	=	**↗**	=	=
Contractile apparatus	TUBA-8	**↘**	**↘**	=	=	**↘**	=
	MyH7B	=	**↘**	=	=	=	=

↗: upregulated; ↘: downregulated; = : not-regulated; Norm: Normalization. Effect of sepsis: sepsis *vs* sham. Effect of landiolol in sepsis: sepsis + landiolol vs sepsis. Effect of sepsis + landiolol: sepsis + landiolol *vs* sham.

**Figure 3:**
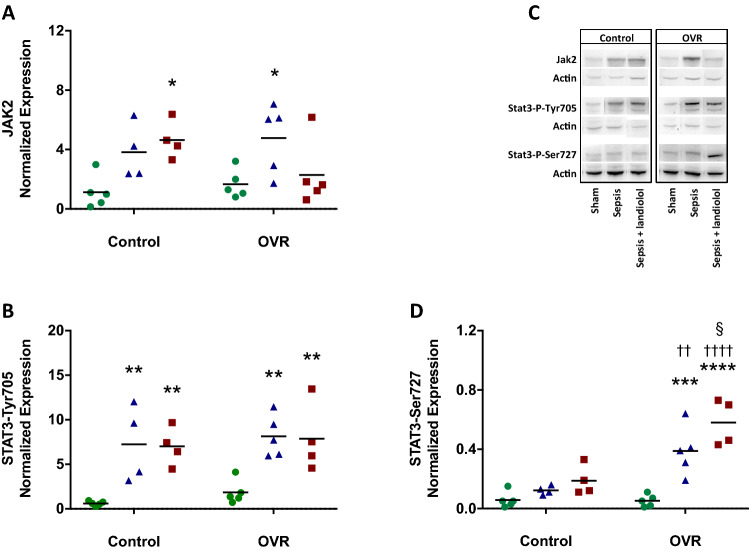
Left ventricular JAK2 expression and STAT3 phosphorylation assessed by western-blot in sham (n = 5), sepsis (n = 4), sepsis plus landiolol (n = 4), OVR-sham (n = 5), OVR-sepsis (n = 5) and OVR-sepsis plus landiolol (n = 4). (**A**) JAK2 expression. (**B**) STAT3-Tyr705 phosphorylation. (**C**) The phosphorylation of STAT3 and the expression of JAK2 were normalized to actin on stripped blot. (**D**) STAT3-Ser727 phosphorylation. *p < 0.05; **p < 0.01; ***p < 0.001; ****p < 0.0001 vs sham and vs OVR-sham, respectively. ^§^p < 0.05 *vs* OVR-sepsis. ^††^p < 0.01; ^††††^p < 0.0001 vs control of corresponding group.

We then checked that JAK2 protein and transcript expression were correlated and looked at the phosphorylation level of STAT3 at its two phosphorylation sites Tyr705 and Ser727 by Western-Blot. The increase in JAK2 protein during sepsis was significant only in OVR females (Fig. 3). Regarding STAT3 phosphorylation, both groups showed an increase at the Tyr705 site during sepsis while only OVR females showed a significant phosphorylation at the Ser727 site (Fig. 3).

**Figure 4:**
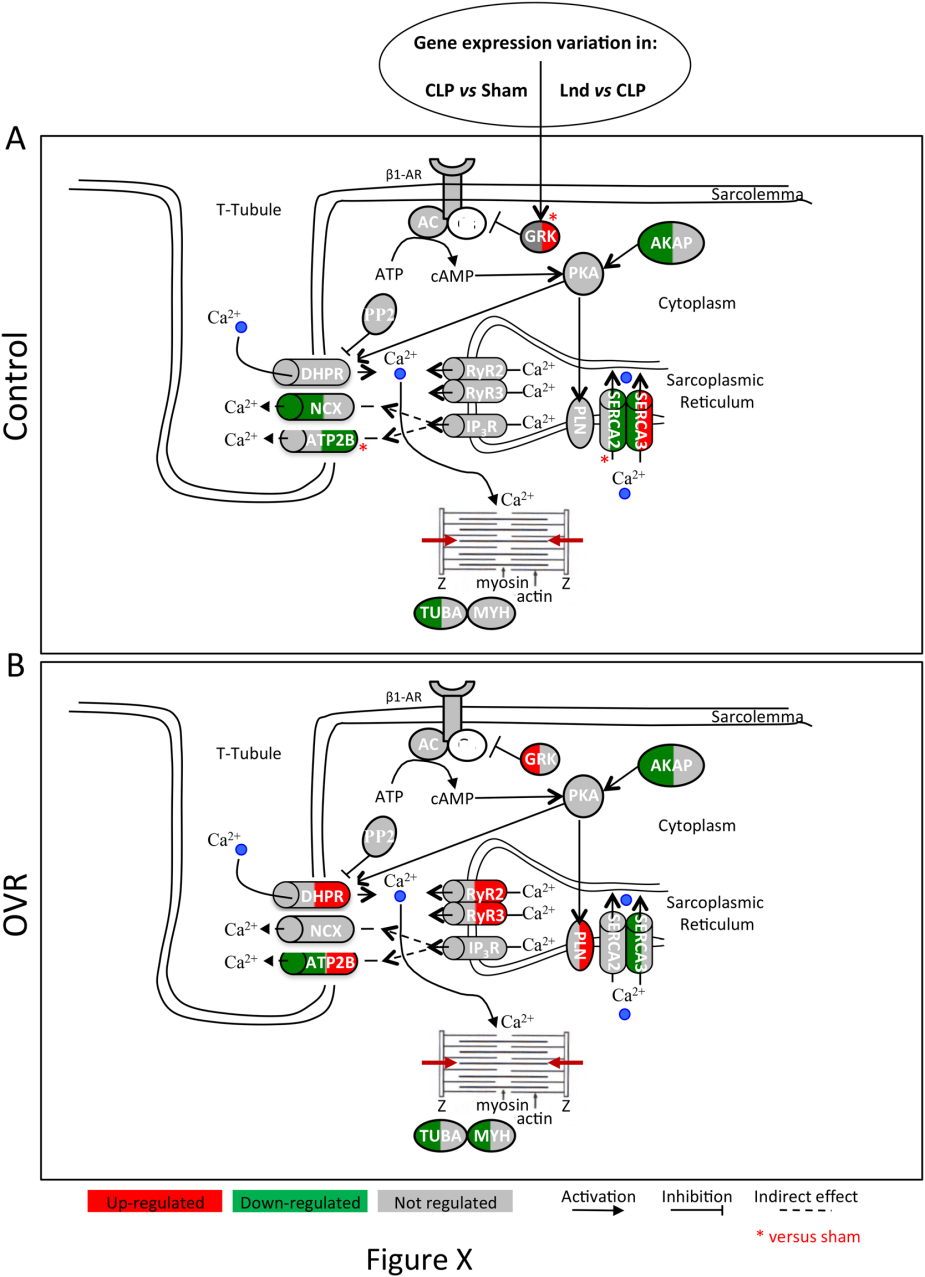
Transcripts regulation by sepsis and landiolol of the adrenergic and excitation/contraction coupling pathways. (**A**) In control females. (**B**) In OVR females. In red: up-regulated, green: down-regulated and grey: not regulated transcripts in sepsis *vs* sham: left part of symbol, in sepsis + landiolol vs sepsis: right part of symbol; *in sepsis + landiolol vs sham.

Regarding transcripts of the adrenergic pathway, sepsis decreased the α-adrenergic receptor (α-AR) and phospholipase C beta 4 (PLCB4) expression and increased G protein-coupled receptor kinase 5 (GRK5) that inhibits adrenergic pathways, only in OVR females (Table 3 and Supplementary Table 2). In addition, sepsis acted differently on genes involved in excitation/contraction coupling. Thus, A-Kinase Anchoring Protein 6 (AKAP6), which binds to the regulatory subunit of protein kinase A (PKA), and tubulin alpha 8 (TUBA8) were decreased in both control and OVR females; however, the inhibition of the β-adrenergic/PKA pathway was potentiated by the increase of GRK5 in OVR females, in which the myosin heavy chain 7B (MYH7B) contractile protein was also decreased. Regarding relaxation, sepsis reduced the ATPase Sarcoplasmic/Endoplasmic Reticulum Ca^2+^ Transporting 3 (SERCA3) expression in both groups and decreased solute carrier family 8-member 1 (SLC8A1) transcript expression, the Na^+^/Ca^2+^ exchanger, in control female and the ATPase plasma membrane Ca^2+^ transporting protein (ATB2B2) expression in OVR female (Fig. 4 and Table 3).

### Effects of landiolol on cardiac function OVR females

Both in control and OVR females, landiolol administration after sepsis did not affect physiological variables (Table 1).

Heart rate (HR) was lowered during landiolol administration in control and OVR females, although the difference did not reach a significant level. In control females, landiolol administered after sepsis decreased systolic blood pressure and mean blood pressure by 27% and 30% respectively (p < 0.01), as compared with sham. In contrast, landiolol administration after sepsis did not affect blood pressure in OVR females (Table 1).

In control females, after sepsis, landiolol decreased the cardiac global performances: CI and SVi were decreased by 44% (p < 0.0001) and 37% (p < 0.01) respectively. Conversely, in OVR females, after sepsis, landiolol improved the impaired diastolic function and the cardiac global performances as EDVi was increased by 33% (p < 0.01) and SVi by 51% (p < 0.05) (Fig. 2). Landiolol did not affect LVEF and LVM in control and OVR females.

In control females, after sepsis, landiolol did not change EB lung tissue concentration. No differences of EB lung tissue concentration were found between OVR-sepsis females and OVR-sepsis plus landiolol females. There were no differences in lactate and procalcitonin plasma concentration between OVR-sepsis females and OVR-sepsis plus landiolol females (Table 2).

Regarding transcripts regulation, landiolol up-regulated the transcript of IL-1b in OVR females and potentiated the sepsis-induced increase of TNFα (Table 3).

Landiolol significantly increased JAK2 protein expression in control females, as compared with sham, while this expression decreased in OVR females being no longer different from sham. While STAT3 phosphorylation at Tyr705 remained high in both groups in the presence of landiolol, the potentiation of phosphorylation at Ser727 site was noted only in OVR females. The phosphorylation status at this site was higher, as compared with control females (Fig. 3).

Regarding excitation/contraction coupling, landiolol increased the expression of GRK5 and maintained the decrease of AKAP6 that could contribute to the inhibition of the adrenergic/PKA pathways in control females and reversed the sepsis-induced elevation of cAMP Responsive Element Modulator (CREM) expression only in OVR females. Moreover, in OVR females, landiolol increased the expression of Calcium Voltage-Gated Channel Subunit Alpha1 C (CACNA1C), Ryanodine Receptor 2 (RYR2) and 3 (RYR3), enhancing calcium entry into the cardiomyocytes in OVR females. At the level of relaxation, in control females, landiolol restored SERCA3 but decreased SERCA2 and ATP2B2 expression, which could contribute to Ca^2+^ efflux inhibition, whereas the Na^+^/Ca^2+^ exchanger remained down-regulated. Conversely, in OVR females, landiolol did not affect the expression of SERCA2, Na^+^/Ca^2+^ exchanger but increased the expression of ATP2B2 (Fig. 4 and Table 3).

### Mortality rates

There were no deaths in the sham control or OVR females before the end of the protocol. In control females, the mortality rate was 12.5% (one animal) after sepsis and 42.8% (three animals) in sepsis + landiolol group. In OVR females, this mortality rate was 0% after sepsis and 14.3% in the OVR sepsis + landiolol group. There was no statistically significant difference between the groups.

## Discussion

There is increasing evidence that sex, and particularly female hormones, affects the response to sepsis^2,4,14^. However, the mechanisms of this phenomenon remained to be demonstrated. To our knowledge, we provide the first study comparing cardiac performances in OVR and control female septic rats. A major point of our approach is the assessment of cardiac function using MRI, thereby providing an in vivo evaluation of cardiac function that includes heart-vessels interactions. Our results highlight disparities between control and OVR female rats. No impairment of cardiac performances after sepsis was observed in control females whereas in OVR females, sepsis decreased SVi and CI related to an EDVi diminution.

Different mechanisms may explain this effect. The alteration in cardiac performance (decrease in SVi and CI) in septic OVR females was associated with a decrease in EDVi without effect on LVEF, DBP and MBP. This suggests a left ventricular diastolic dysfunction with a defect of filling during the diastole, while the venous return and the myocardial contractility were preserved. In our previous study^8^, we showed that this effect was obtained in males, at variance with females.

Other experimental models have found improved survival in female mice after CLP compared to males^10,14^. This is in accordance with our present data in control females: in OVR septic females, the hemodynamic profile seemed to be close to that of septic males.

This suggests a protective effect of female hormones in sepsis, especially at the cardiovascular level. Female hormones have a protective effect on cardiac functions and cardiovascular response during major inflammation^4^. Experimental administration of 17-β-œstradiol improved functional recovery after ischemia–reperfusion in rats^9^. In an endotoxin-induced sepsis model, administration of 17-β-œstradiol mitigated septic cardiac dysfunction^15^. Different mechanisms could explain the protective effect of female hormones in sepsis; an immunomodulatory effect of oestrogens has been reported in various models^16^. As these works have focus on oestrogens effects, our model, however, removes all ovarian hormones. Therefore, the measured effects could be imputed either to oestrogens or progesterone, or the combination of both. Further investigations are needed to determine the role of each female hormone in the protective processes of the cardiac function in sepsis. Short acting β-blocker treatment may be a promising therapeutic approach in sepsis. Its efficacy has been shown in male rats septic models^8,17^. Our study confirms these findings in OVR females, since landiolol administration during sepsis improved SVi and CI with an increase in EDVi. The present report suggests that the lack of effect of landiolol in females was mediated by female hormones. Thus, landiolol improved cardiac performance in conditions suggesting a reduced exposure to female hormones during sepsis.

In the second part of our study, we looked for the underlying mechanism of changes in hemodynamic profiles. We previously identified, in a transcriptomic study, major differences in sepsis-induced deregulation of gene expression in male and female rats and confirmed the benefit of landiolol intake on cardiac function in male^13^. Our current results highlight a similar gene expression profile in OVR females to that found in males. In particular, septic OVR females showed a more pronounced inflammatory and apoptotic profile, including the JAK/STAT pathway. In addition, decreased expression of genes involved in the α- and β-adrenergic pathways, as well as in Ca^2+^ efflux, could explain the decreased blood pressure and diastolic dysfunction observed in OVR females. In these animals, landiolol improved cardiac performances, which was supported by the restoration of JAK2 expression, and the overexpression of genes responsible for Ca^2+^ influx in cardiac cells. Conversely, the deleterious effect of landiolol in control females may be explained by the overexpression of JAK2, associated with a decreased expression of genes involved in Ca^2+^ efflux and β-adrenergic regulation. These results are in line with findings on the human cardiopathy during sepsis^18^. In septic rat, dysregulation of genes involved in α-adrenergic, β-adrenergic and Ca^2+^ cycling pathway was associated with reduced survival^19^. These transcriptomic regulations reduce the L-type calcium current^20^ and increase the cytosolic calcium that lead to failure of diastolic relaxation, and decrease the calcium concentration in the sarcoplasmic reticulum, which affect systolic contraction as previously described in septic cardiomyopathy^21^.

In contrast to previous studies^8,22^, we did not show any significant decrease in HR during treatment with landiolol, regardless of hormonal status. This lack of difference may be explained by a relatively low number of analysed rats, which probably was a drawback for statistical correlations. Another possible explanation could be a sub-optimal dosage of landiolol. This, however, seems unlikely since we used the same dosage as that previously used to obtain a decrease in HR^8^.

During sepsis, there are structural and functional changes in the endothelium responsible for endothelial dysfunction and microcirculation disorders^23,24^, resulting in higher permeability and oedema. Estradiol administration in male rats and in OVR females has been shown to improve intestinal microcirculation during sepsis^25^. However, we found no significant differences in EB lung tissue concentration between the various groups, although there was a trend toward an increased permeability in OVR females. Further investigations are needed to define the effects of female hormones on pulmonary endothelium and microcirculation during sepsis.

Our study has several limitations. Our experimental model of sepsis, CLP, is considered as the reference method^26^, but the main drawback of this method is its lack of reproducibility^27,28^. We attempted to reproduce the experimental conditions obtained in our previous study. However, we reduced the vascular filling performed during the 18 h after CLP, to better mimic the initial phase of human septic shock. This could have result in differences in the severity of sepsis compare to our previous work, and partially explain the differences we found in genes expression in control females rats following CLP and landiolol, compare to our previous study. This also resulted in an increased mortality in our septic animals. Nevertheless, we found deleterious consequences and more impaired cardiac performance in the rats assessed in the group with the highest mortality (sepsis plus landiolol females). Another limitation was that our study did not assess the baseline response to landiolol. Landiolol is hydrolysed to an inactivate metabolite by esterases, which activities are higher in female rats than in male rats^29^. Whether this activity can be affected by OVR is unknown. Response to general anesthesia may also be sex-mediated^30^, and therefore affected by OVR. Finally, the plasma concentrations of female sex hormones are likely to vary depending on oestrus cycle in females, and this cycle was not considered during sepsis.

## Conclusions

Our model confirms that cardiac function was more impaired in septic OVR females, as compared with control females, suggesting a protective effect of female hormones on septic cardiac dysfunction. This difference in cardiac performances was associated with a more pronounced activation of inflammation pathways and inhibition of adrenergic pathways in OVR females. Landiolol, while it produced deleterious effects in control females, prevented cardiac dysfunction in OVR females due to an improvement in diastolic function, thereby mimicking its known effect in males. This was supported by an overexpression of genes involved in the Ca^2+^ influx in OVR females while an inactivation of the β-adrenergic and the Ca^2+^ efflux pathways was observed in control females. Interactions between sex hormones and β-blocker deserve further investigations on larger series to better understand the cellular mechanisms leading to a protective effect in sepsis.

## Supplementary Information

Supplementary Information.
