# Supplementary material for: Female hormones prevent sepsis-induced cardiac dysfunction: an experimental randomized study
Source: Sci Rep. 2022 Mar 23;12:4939. doi: 10.1038/s41598-022-08889-4 (PMC8943058; doi:10.1038/s41598-022-08889-4)
Supplement: Supplementary file 1 — Supplementary Information. [file 41598_2022_8889_MOESM1_ESM.docx]

**Bilateral ovariectomy procedure**

The surgery was performed under general anesthesia combining an inhalated anesthesia with halogenated gas (isoflurane) and morphine (buprenorphine 0.05 mg/kg, sub-cutaneous). After induction with isoflurane 5% in an induction chamber, anesthesia was maintain using a facial mask. The rats were kept in spontaneous ventilation using 2% isoflurane combined with 1.5L /min of oxygen. The depth of the anesthesia was checked by the disappearance of the leg withdrawal reflex. The rats were placed on a hot plate for the duration of the surgery to prevent hypothermia.

After mowing and skin disinfection, a 2 cm transverse skin incision was made on the animal's back, just below the rib cage. After dissection of the subcutaneous tissue, a small muscle incision was made on each side of the midline, approximately 0.5 cm from the latter. The peri-ovarian fat was then identified and carefully exteriorized outside the abdominal cavity. The ovary and oviduct were located within the fat. For control procedures, the ovaries were simply replaced in the abdominal cavity. For bilateral ovariectomy procedures, a ligature was performed at the level of the oviduct and the 2 ovaries were removed. The uterus and the remaining part of the oviduct were then replaced in the abdominal cavity and the closure was done by suturing the muscular planes and then the skin. Perioperative rehydration by injection of 1 ml of 0.9% NaCl subcutaneously was performed at the end of the intervention.

**MRI Assessment of In Vivo Cardiac Function**

Cardiac MRI was performed 18 hours after the induction of sepsis, immediately after placement of the arterial catheter. The images were acquired on a Bruker Biospec Avance 4.7 T / 30 system (equipped with a horizontal magnet of 4.7 Tesla) with the exception of one animal for which the images were acquired on a Bruker Pharmascan 7 system .0 T / 16 (fitted with a 7.0 Tesla horizontal magnet). The rat was placed in a prone position on a surface probe allowing the reception of radio frequency signals and was then placed into the magnet. A volume proton resonator (60 mm in diameter and 80 mm in homogeneous length) was used as a homogeneous source of excitation radio frequency. During images acquisition, anesthesia was maintain with isoflurane combined with 1.5 L / min of oxygen, via a facial mask, at concentrations between 1.8 and 2% allowing the maintenance of spontaneous ventilation. A heating blanket was placed over the animal to prevent hypothermia. The electrocardiogram (ECG) was monitored by two electrodes placed subcutaneously, and the respiratory rate was monitored by a ventral balloon. The servo control of magnetic resonance acquisition by the ECG and respiration was carried out using a synchronization unit from the company Rapid Biomedical and the Acq Knowledge interface.

The cine-MRI sequence was used to acquire the short-axis and long-axis orientation sections 2 chambers and 4 chambers (FLASH, Field Of View 4 x 4 cm2, cutting thickness 2 mm, size of the matrix 128 x 128, repetition time TR = 5.1 ms, echo time TE = 1.2 ms, approximately 45 phases per cardiac cycle). Images acquisition was synchronized with the ECG.

Image processing was carried out using post-processing tools developed in the laboratory operating in the IDL environment (Interactive Data Language, Excelis, United States) without having knowledge of the groups to which the animals belonged. The determination of the left ventricular volumes used the ellipsoid model; where the left ventricle is assimilated to an ellipsoid of revolution. The endocardial and epicardial areas were drawn manually on the images of the strict minor axis section and the ventricular length on the strict 4-chamber major axis section; in systole and diastole.

**Biochemical analyses in plasma**

Plasma lactate concentration was measured using the Lactate assay kit II (Sigma-Aldrich, St. Louis, MO, USA) and plasma procalcitonin concentration was determined using the PCT BioAssay™ ELISA Kit (US Biological, Salem, MA, USA) according to the manufacturer**’**s instructions.

**Supplemental figure 1: Flow-Chart. EB:** Evans Blue experiments


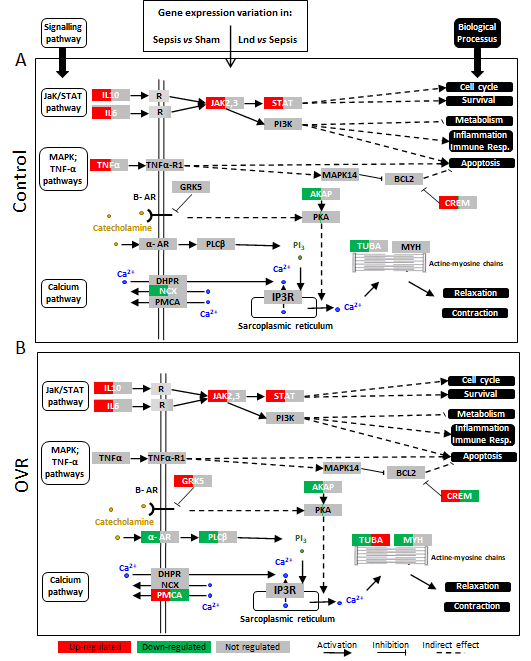


**Supplemental figure 2: Sepsis-induced gene expression variation in selected signalling pathways.** For each gene, the left part corresponds to the variation in expression in the sepsis group, the right part to that in the sepsis + landiolol group. Red color represents upregulated gene; Green color, down-regulated gene; and Grey color, not regulated gene. On the right part of the figure, biological processes impacted by the deregulated genes are indicated.

**Supplemental Table 1: qPCR**

|  | Non OVR | | | |  | OVR | | | |
| --- | --- | --- | --- | --- | --- | --- | --- | --- | --- |
|  | Sham  (n=5) | CLP  (n=4) | CLP + landiolol  (n=4) |  | | | Sham  (n=5) | CLP  (n=5) | CLP + landiolol (n=5) |
| *Pro-inflammatory Molecules* | | | | | | | | | |
| IL-6 | 15.7 ± 0.2 | 4.5 ± 1.1^****^ | 4.0 ± 1.8^****^ |  | | | 15.8 ± 0.3 | 4.0 ± 0.8^****^ | 3.0 ± 1.2^****^ |
| TNFA | 9.8 ± 0.3 | 8.1 ± 1.2^*^ | 7.1 ± 0.9^***^ |  | | | 8.8 ± 0.3 | 7.7 ± 0.2 | 7.5 ± 1.3 |
| TNFRSF26 | 6.8 ± 0.6 | 6.2 ± 0.3 | 6.0 ± 0.6 |  | | | 6.6 ± 0.5 | 6.4 ± 0.4 | 5.9 ± 0.7 |
| JAK2 | 4.3 ± 0.5 | 3.0 ± 0.8^**^ | 2.4 ± 0.3^****^ |  | | | 3.9 ± 0.5 | 1.9 ± 0.1^****, ††^ | 2.2 ± 0.5^****^ |
| STAT3 | 1.9 ± 0.6 | 1.0 ± 0.4^**^ | 0.7 ± 0.4^***^ |  | | | 1.6 ± 0.4 | 0.1 ± 0.2^****, ††^ | 0.5 ± 0.4^***^ |
| IL-1B | 7.7 ± 1.1 | 5.1 ± 1.2^***^ | 4.4 ± 0.5^****^ |  | | | 6.8 ± 0.7 | 5.6 ± 0.6 | 4.1 ± 1.2^***, §^ |
| IL-18 | 7.6 ± 0.3 | 5.8 ± 0.1^****^ | 5.5 ± 0.3^****^ |  | | | 6.8 ± 0.5^†††^ | 5.7 ± 0.2^****^ | 5.3 ± 0.3^****^ |
| AKT2 | 2.3 ± 0.5 | 2.8 ± 0.4 | 2.8 ± 0.5 |  | | | 2.1 ± 0.3 | 2.6 ± 0.1 | 2.4 ± 0.2 |
| PPP2ACA | 0.6 ± 0.2 | 1.0 ± 1.2 | 0.6 ± 0.6 |  | | | 0.3 ± 0.3 | 0.1 ± 0.4 | 0.1 ± 0.3 |
| NFKB1 | 4.1 ± 0.4 | 3.9 ± 0.4 | 3.8 ± 0.2 |  | | | 3.8 ± 0.2 | 3.9 ± 0.1 | 3.6 ± 0.4 |
| *Anti-inflammatory Molecules* | | | | | | | | | |
| IL-10 | 13.4± 1.4 | 9.1 ± 0.5^****^ | 9.5 ± 0.8^****^ |  | | | 12.9 ± 0.4 | 9.2 ± 0.6^****^ | 9.1 ± 0.8^****^ |
| *Apoptosis* | | | | | | | | | |
| BCL2 | 5.0 ± 0.6 | 5.5 ± 0.9 | 5.2 ± 0.4 |  | | | 4.6 ± 0.5 | 5.1 ± 0.3 | 4.7 ± 0.6 |
| CREM | 3.3 ± 0.5 | 2.2 ± 0.6^**^ | 2.9 ± 0.6 |  | | | 3.7 ± 0.2 | 2.2 ± 0.4^***^ | 3.1 ± 0.6^§^ |
| MAPK14 | 3.3 ± 0.5 | 3.2 ± 0.3 | 3.4 ± 0.4 |  | | | 3.0 ± 0.4 | 2.0 ± 0.3^**, †††^ | 2.6 ± 0.5^†^ |
| *Adrenegic Pathway* | | | | | | | | | |
| ADRA1A | 3.2 ± 0.7 | 3.8 ± 0.6 | 3.9 ± 0.8 |  | | | 2.8 ± 0.3 | 4.0 ± 0.4^*^ | 3.6 ± 0.7 |
| PLCB4 | 2.2 ± 0.5 | 2.6 ± 0.3 | 2.6 ± 0.3 |  | | | 2.1 ± 0.4 | 3.2 ± 0.2^***^ | 2.6 ± 0.5 |
| PRKACA | 0.6 ± 0.5 | 1.5 ± 1.2 | 1.4 ± 0.7 |  | | | 0.3 ± 0.4 | 1.3 ± 0.3 | 0.8 ± 0.4 |
| GRK5 | 4.3 ± 0.7 | 3.7 ± 0.3 | 3.4 ± 0.4^*^ |  | | | 3.5 ± 0.5^†^ | 2.6 ± 0.1^**, ††^ | 3.2 ± 0.3 |
| ADCY9 | 4.8 ± 0.6 | 5.1 ± 0.6 | 5.2 ± 0.5 |  | | | 4.5 ± 0.5 | 5.1 ± 0.2 | 5.0 ± 0.3 |
| *Calcium Signaling* | | | | | | | | | |
| AKAP6 | 2.0 ± 0.5 | 2.9 ± 0.5^*^ | 2.9 ± 0.6^*^ |  | | | 2.0 ± 0.3 | 3.0 ± 0.2^**^ | 2.3 ± 0.7 |
| ITPR1 | 4.0 ± 0.6 | 3.7 ± 0.6 | 3.7 ± 0.4 |  | | | 3.4 ± 0.3 | 3.5 ± 0.8 | 3.8 ± 0.3 |
| SLC8A1 | 1.6 ± 0.4 | 2.9 ± 1.3^*^ | 2.8 ± 1.1 |  | | | 1.6 ± 0.3 | 2.5 ± 0.7 | 2.3 ± 0.3 |
| ATP2B2 | 6.2 ± 0.9 | 7.4 ± 0.4 | 8.1 ± 0.7^**^ |  | | | 7.0 ± 1.3 | 8.7 ± 0.4^*^ | 7.1 ± 0.8^§^ |
| RYR2 | -0.3 ± 0.3 | 0.1 ± 0.3 | 0.3 ± 0.6 |  | | | -0.5 ± 0.3 | -0.1 ± 0.5 | -0.9 ± 0.3^§§, †††^ |
| RYR3 | 4.6 ± 0.3 | 4.5 ± 0.3 | 4.0 ± 0.5 |  | | | 4.1 ± 0.2 | 4.2 ± 0.3 | 3.5 ± 0.4^§^ |
| SERCA2 | -4.3 ± 0.4 | -3.9 ± 0.5 | -2.9 ± 1.5^*^ |  | | | -4.4 ± 0.3 | -4.4 ± 0.3 | -4.9 ± 0.3^†††^ |
| SERCA3 | 7.3 ± 0.2 | 8.3 ± 0.6^**^ | 7.5 ± 0.3^§^ |  | | | 7.1 ± 0.5 | 7.9 ± 0.1^*^ | 7.2 ± 0.4 |
| CACNA1C | 2.6 ± 0.6 | 2.8 ± 0.5 | 3.0 ± 0.5 |  | | | 2.2 ± 0.3 | 2.8 ± 0.5 | 2.0 ± 0.3^§, ††^ |
| PLN | -2.8 ± 0.3 | -2.4 ± 0.4 | -2.2 ± 0.9 |  | | | -3.1 ± 0.3 | -2.6 ± 0.2 | -3.4 ± 0.4^§, ††^ |
| *Contractil Apparatus* | | | | | | | | | |
| TUBA8 | 1.2 ± 0.5 | 3.0 ± 1.6^*^ | 3.1 ± 0.7^*^ |  | | | 1.4 ± 0.7 | 2.9 ± 0.4^*^ | 2.2 ± 0.9 |
| MYH7B | 4.6 ± 0.3 | 4.9 ± 1.1 | 5.4 ± 0.5 |  | | | 4.6 ± 0.3 | 6.0 ± 0.4^**, †^ | 5.5 ± 0.8 |
|  |  |  |  |  | | |  |  |  |

**Supplemental Table 1**: Effects of CLP and landiolol on expression levels of different genes after 18 hours. Data are expressed as average delta cycle threshold (deltaCT) and RPL32 is the housekeeping gene; the lower the deltaCT value, higher the expression. Data are expressed as means ± standard error (SD).

^*^ p < 0.05, ^**^ p < 0.01, ^***^ p < 0.001 vs. sham Non OVR and vs. sham OVR respectively;

^§^ p < 0.05 vs. CLP female;

^†^ p < 0.05 vs. Non OVR of the equivalent group.

**Supplemental Table 2 : Primers**

| **Gene** | **Forward** | **Reverse** |
| --- | --- | --- |
| RPL32 | TGTCCTCTAAGAACCGAAAAGCC | CGTTGGGATTGGTGACTCTGA |
| TUBA8  MYH7B  AKAP6 | TATACGGTGGGCAAGGAGAG  GCAGGACCTAGTGGACAAGC  GCCGAGAAACAGCTCCAATA | CCCACCAAAACTGTGGAAGA  GCTCCTCAGCGTCGTCTAAC  ACCTCAGAATGGAGGCCACT |
| TNFA | ACCACGCTCTTCTGTCTACTG | CTTGGTGGTTTGCTACGAC |
| IL-1B | CTGTGACTCGTGGGATGATG | GGGATTTTGTCGTTGCTTGT |
| IL-18 | CAAAAGAAACCCGCCTGTGT | TCACAGCCAGTCCTCTTACTTCAC |
| JAK2 | CAAAGTCTTGCCACAGGACA | CCAAAGCTCCACACATCTGA |
| IL-6 | AGCCAGAGTCATTCAGAGCA | AGAGCATTGGAAGTTGGGGT |
| IL-10 | CCTTACTGCAGGACTTTAAGGGTTA | TTTCTGGGCCATGGTTCTCT |
| NPR3 | CTCCAAACAGTCACCCTGC | GCAAAGCCAGAACGTAGAG |
| AKT2 | AGCATCGGTTCTTCCTCAGC | GCGGTGAATTCATCATCAAA |
| BCL2 | TGAACCGGCATCTGCACAC | CGTCTTCAGAGACAGCCAGGAG |
| PPP2CA | GTTGGTGTCCAGAGCTCACC | CAGCTTGGTTACCACAACGA |
| NFKB1 | ATGACCTGGACGACTCTTGG | GGCTCATATGGTTTCCCATT |
| ADRA1A | CGAGTCTACGTAGTAGCC | GTCTTGGCAGCTTTCTTC |
| PLCB4 | AGCTGAAGCTCTCCCATGAC | CCCGTTCTGCCTTATTCTTG |
| CREM  ITPR1  PRKACA  GRK5  SLC8A1  ATP2B2  STAT3  TNFRSF26  MAPK14  ADCY9  RYR2  RYR3  SERCA2  SERCA3  CACNA1C  PLN | AGTCCCCAGCAACTAGCAGA  TCGATATCATCACAGCCCTC  TCTTCGCTGACCAGCCTATC  AAGAGGCACCCCTTCTTCAG  TTGTCGCTCTTGGAACCTCA  GCATCTTCATAGGCCTGGGA  TTCGGAAAGTATTGTCGCCC  TGGAGATGTGCCATGATTGC  GCCCAGATGCCGAAGATG  TCACACATCACCTCCGAGTT  GCTGAACTATTTTGCTCGCAAC  TGAGCCGGATATGAAGTGTG  ATTGTTCGAAGTCTGCCTTCTGTGG  GTATGACGCTGAGGGACCAC  TTGACAATGTTCTGGCAGCC  TACCTTACTCGCTCGGCTATC | TCTTCTTCCTGCGACACTCC  GATGGCCAGGAGTAGCTTTG  CGAAGCAGGTCCTTCAAGTC  CTATGTCCAGCACATCCTTG  TTGCTTCCGGTGACATTGC  CTTCTGTGTTAGCCGCCCT  AATGGTATTGCTGCAGGTCG  TGCTTGGAGTCACACACAGT  CCAGCATCTTTTCCAGCAGG  GTTACAGTAGTGCACGACGG  CCACAGAAGAAGTGGAGACCT  CCAGCGGGATCTTCAATTTC  CATAGGTTGATCCAGTTATGGTAAA  GGAAGCGTGACTCAAAGACC  TCTGGCCACCCCTCGA  CAGAAGCATCACAATGATGCAG |


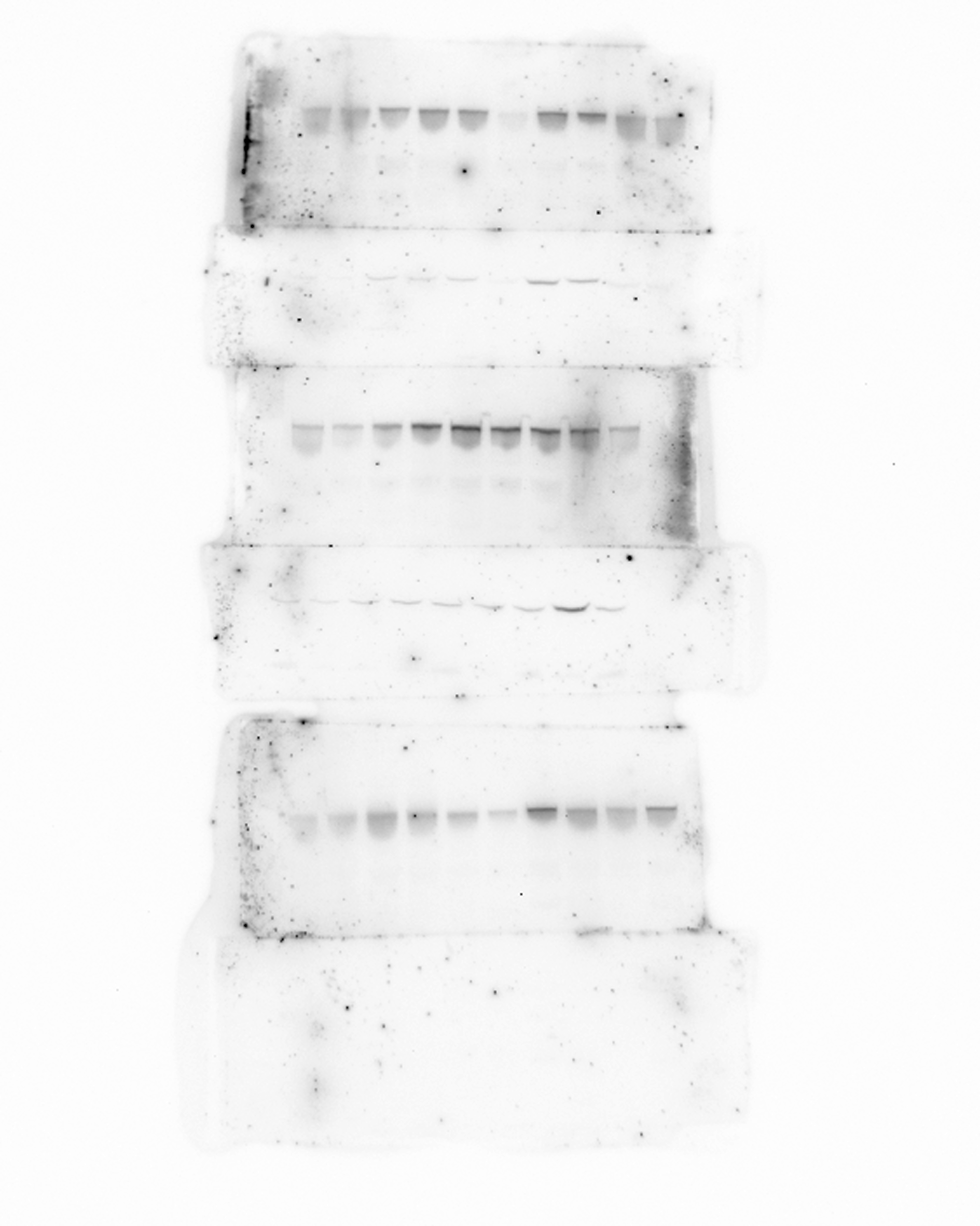

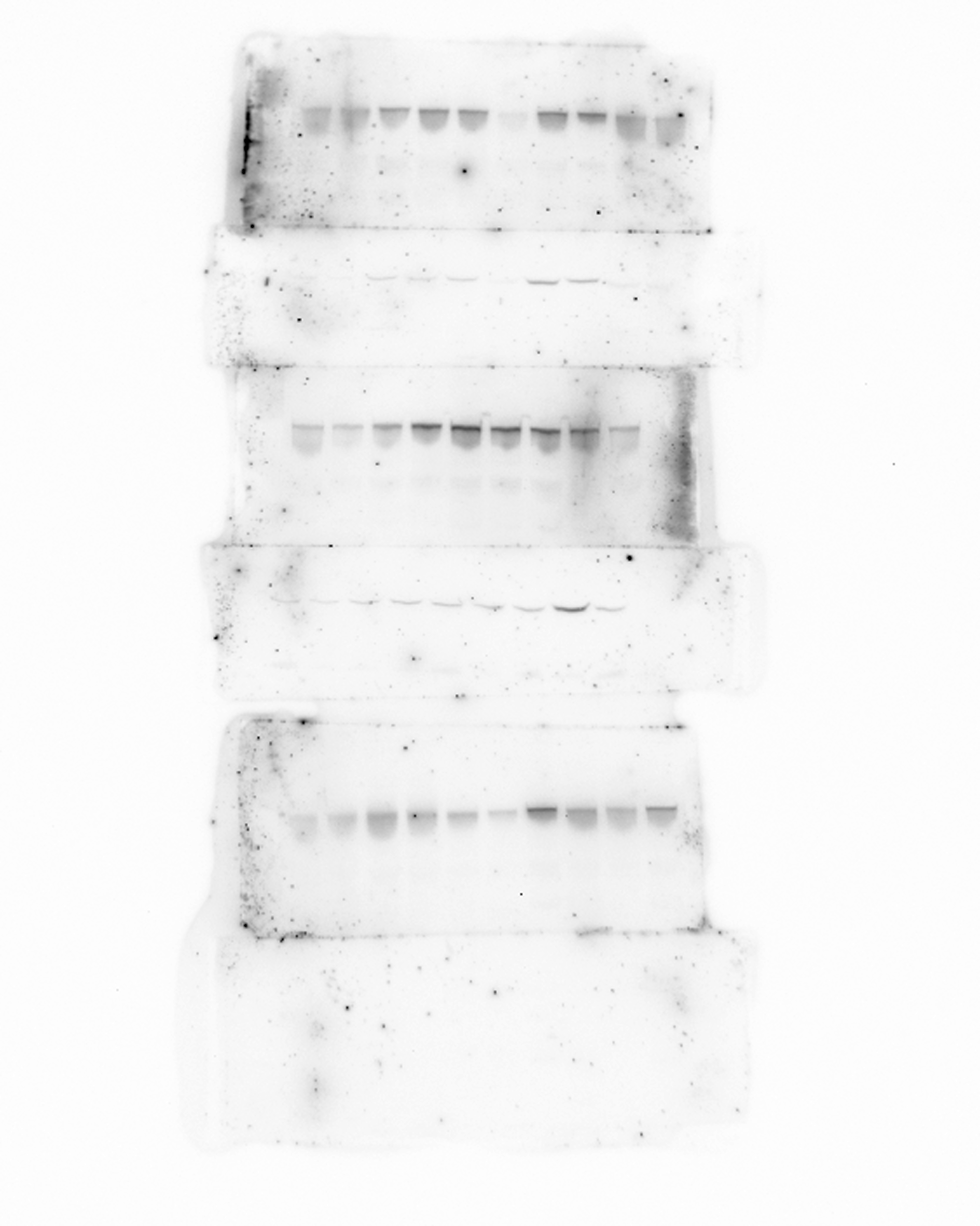

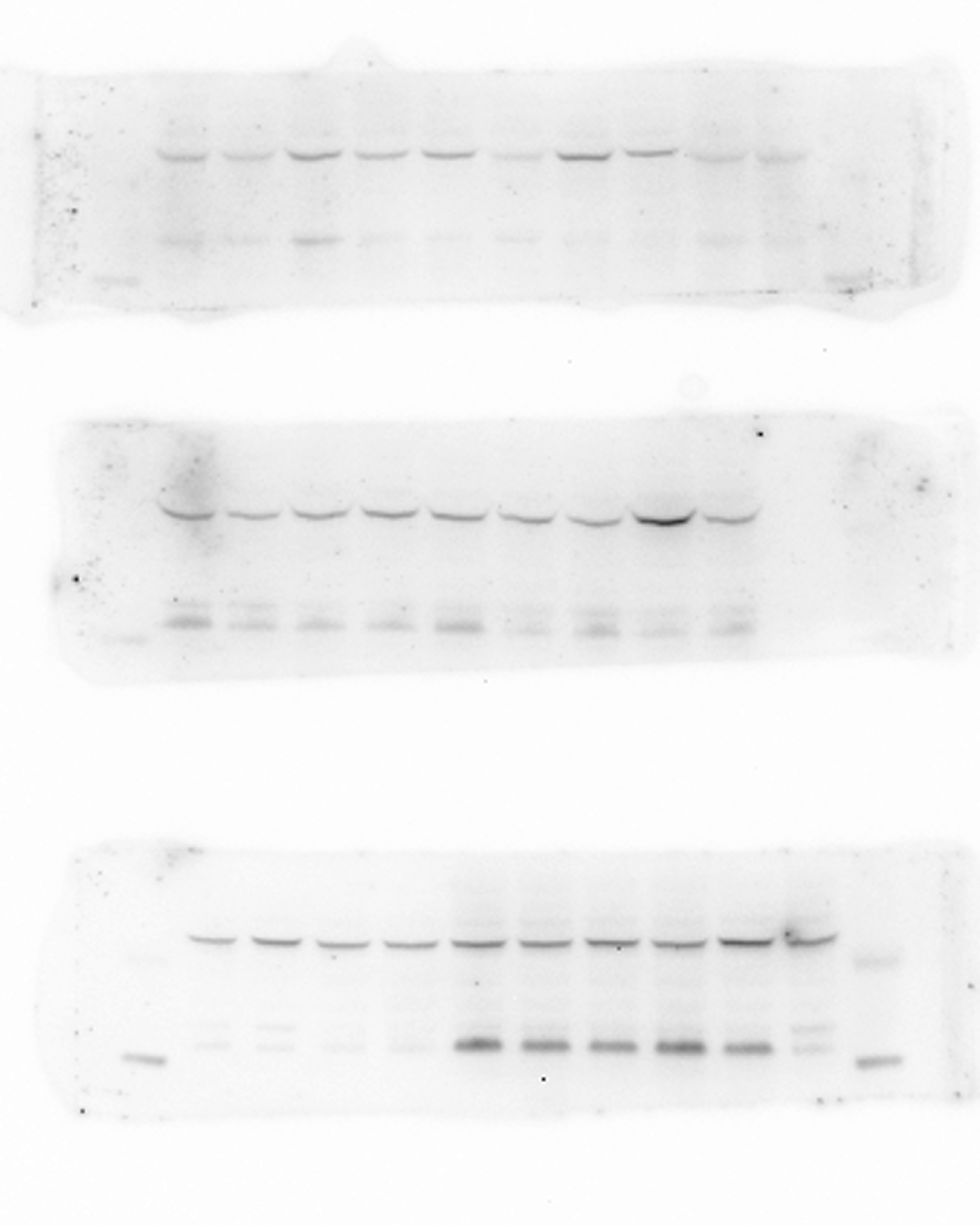

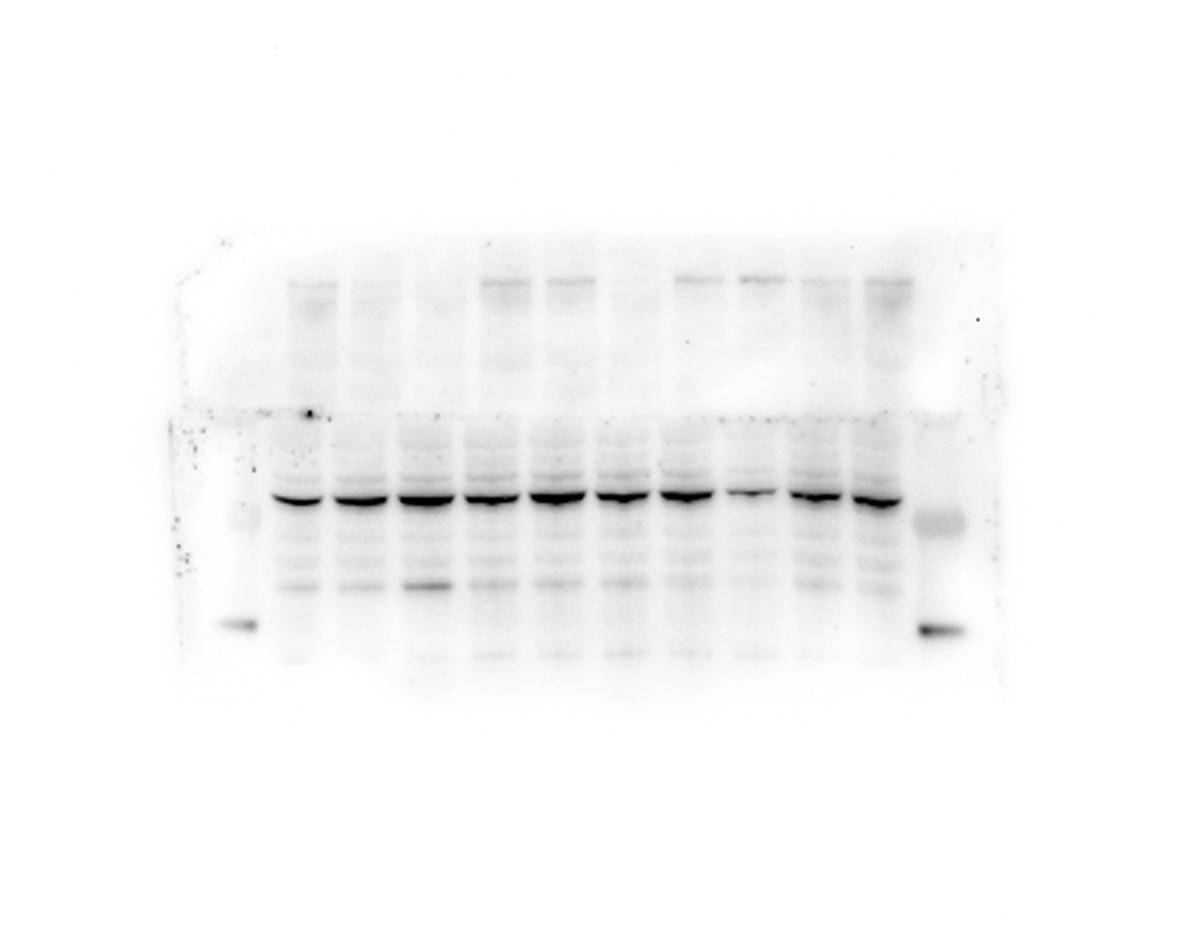

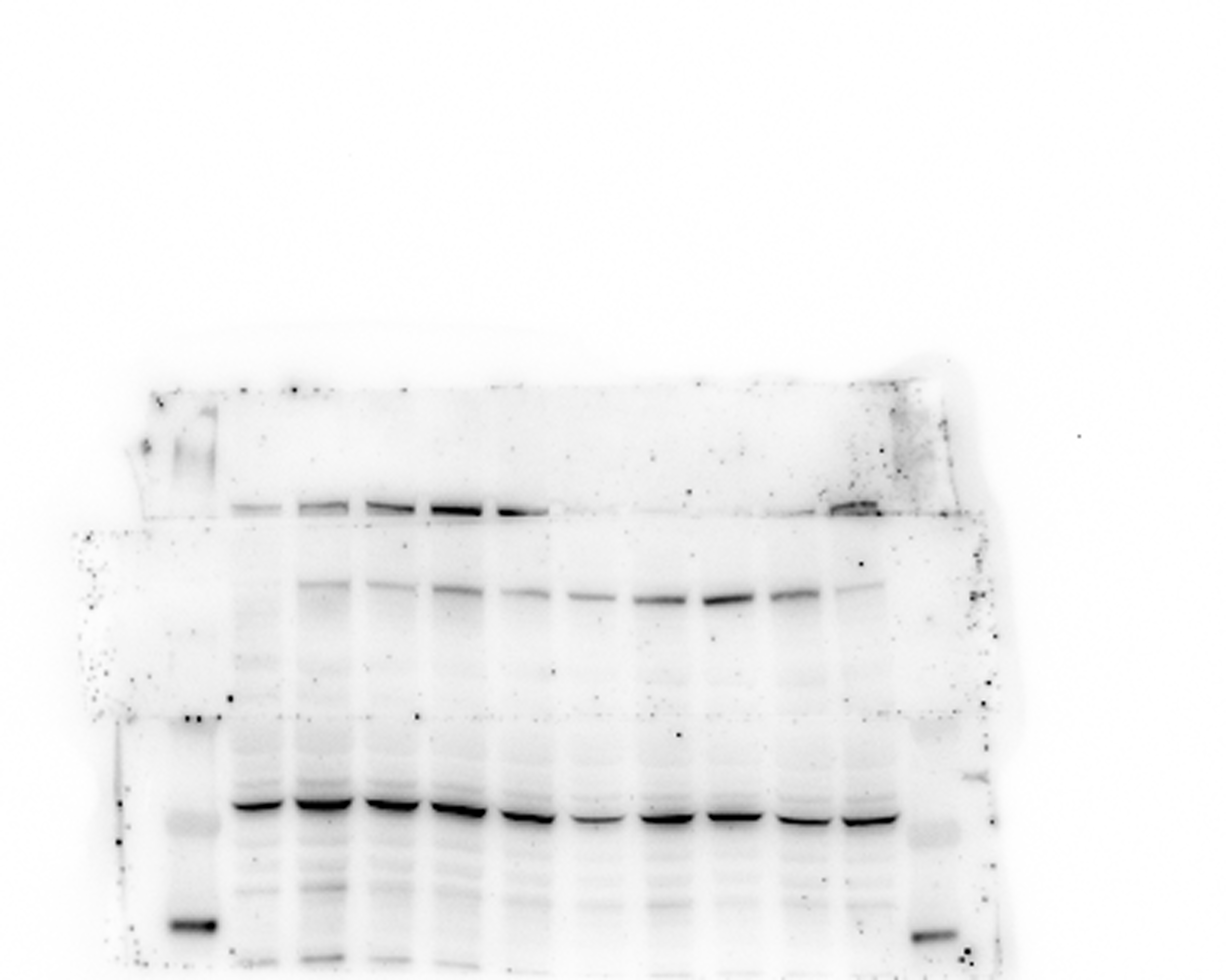

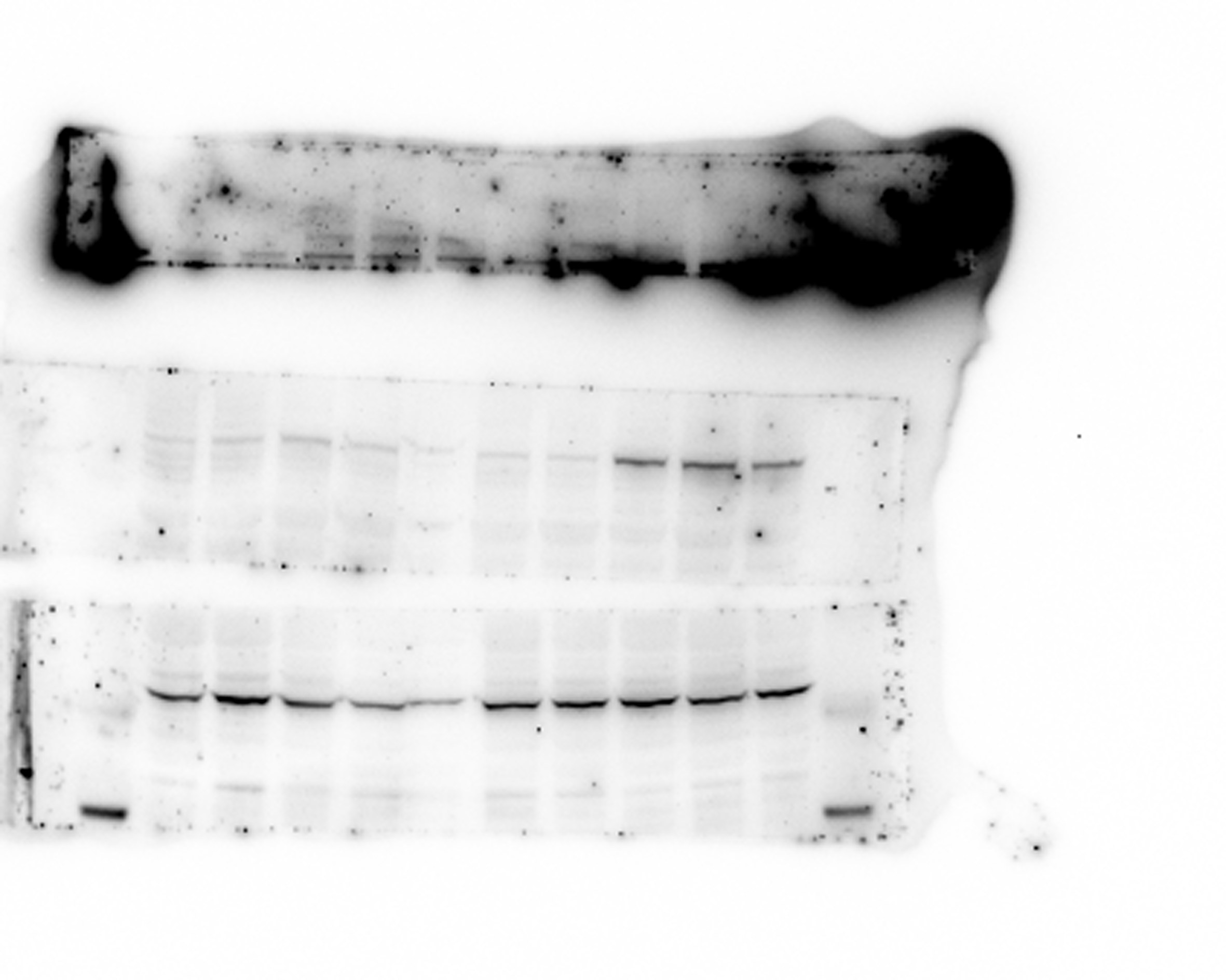

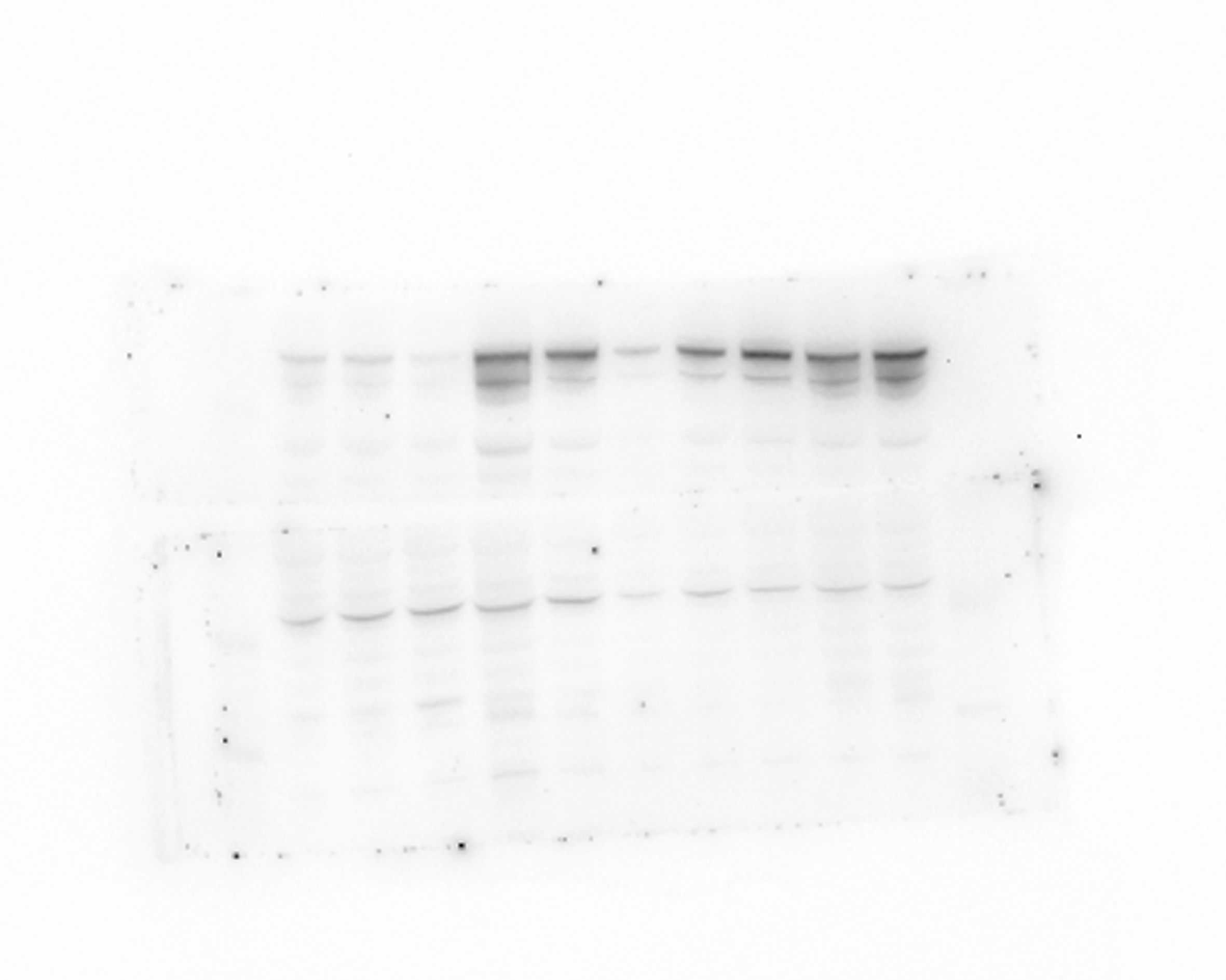

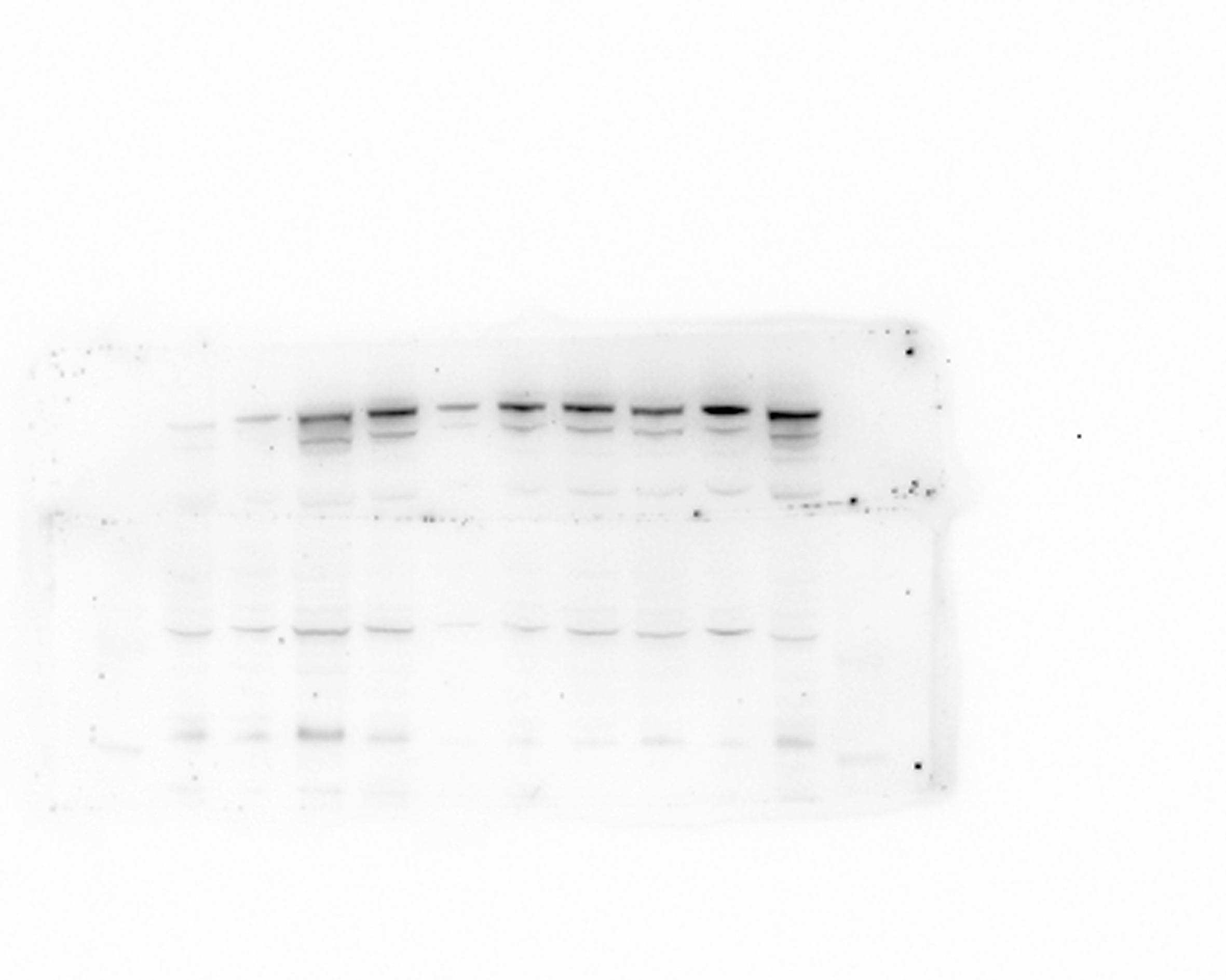

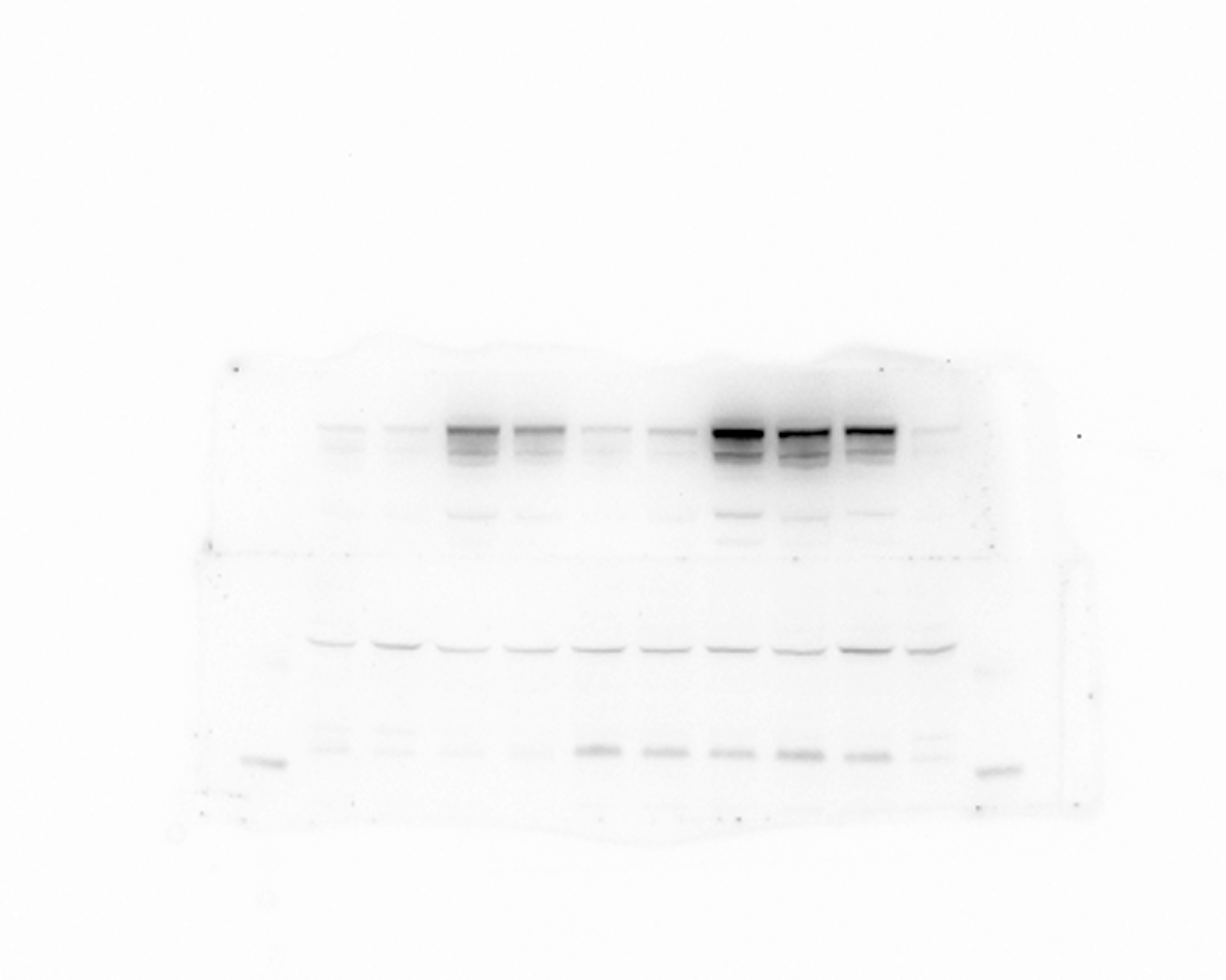


**Stat3-P-Tyr705**

86 kD

79 kD

45 kD

**Actin**

sham

sham

1 **2** 3

1 **2** 3

sepsis

1 **2** 3 4

sepsis+Lnd

OVR

sham

1 **2** 3

1 2 2 **3**

**1** 2 3

OVR

sepsis+Lnd

OVR

sepsis

sham

4 5

OVR

sham

4 5

4 5

sepsis

OVR sepsis

4 5

5 5

OVR

sepsis+Lnd

**Stat3-P-Tyr727**

**Actin**

45 kD

M

M

M

M

M

M

M

M

86 kD

sham

1 **2** 3

1 **2** 3

sepsis

**1** 2 3 4

sepsis+Lnd

**1** 2 3

OVR

sepsis

1 **2** 3 4

OVR

sepsis+Lnd

OVR

sham

1 2 **3**

M

M

M

sham

4 5

OVR

sham

4 5

sepsis

OVR sepsis

4 5

4 4 5

OVR sepsis+Lnd

45 kD

45 kD

86 kD

79 kD

86 kD

79 kD

86 kD

86 kD

45 kD

45 kD

A

B

**Jak2**

**1** 2 3

**1** 2 3

**1** 2 3 4

sham

sepsis

sepsis+Lnd

M

125 kD

C

**Actin**

45 kD


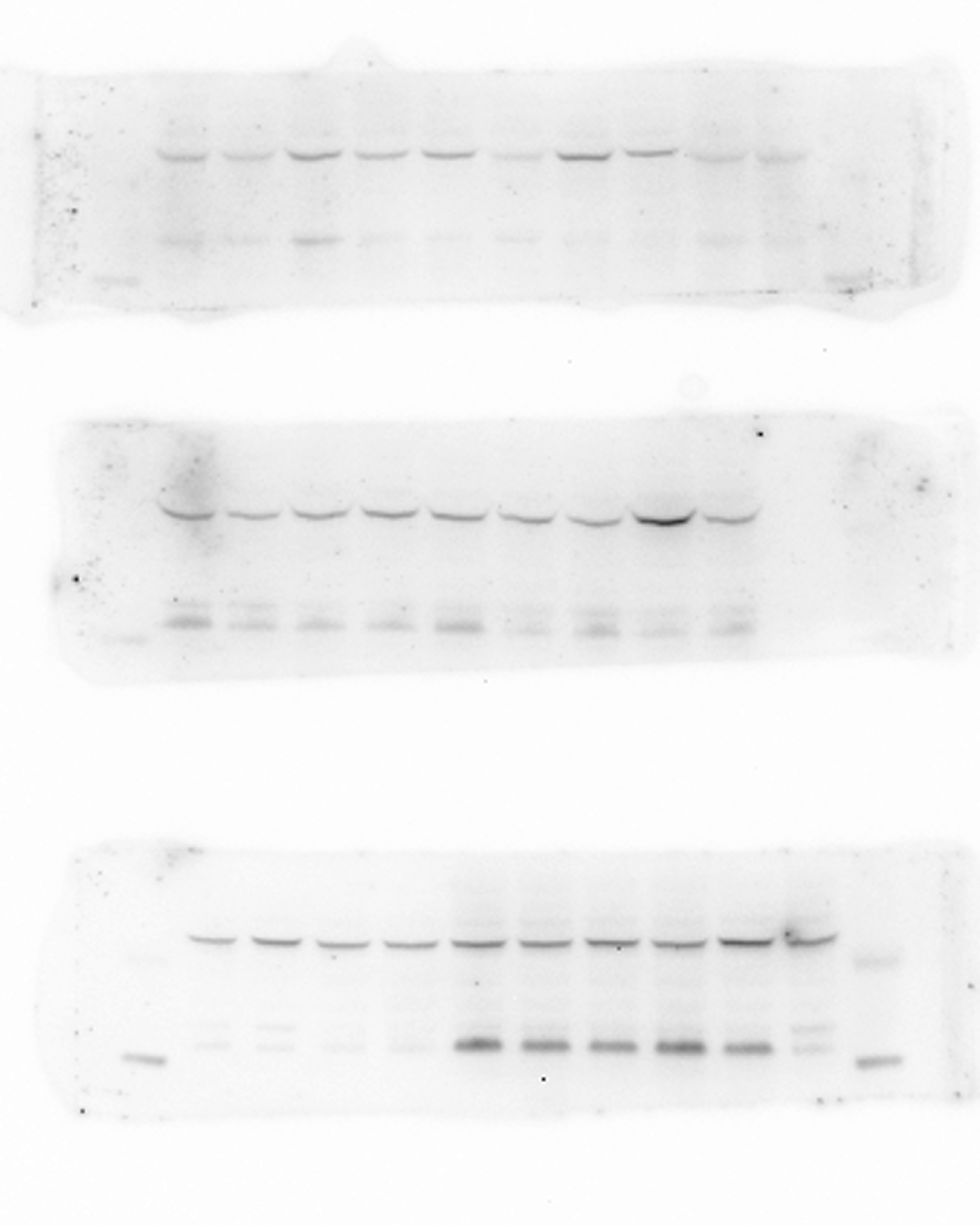

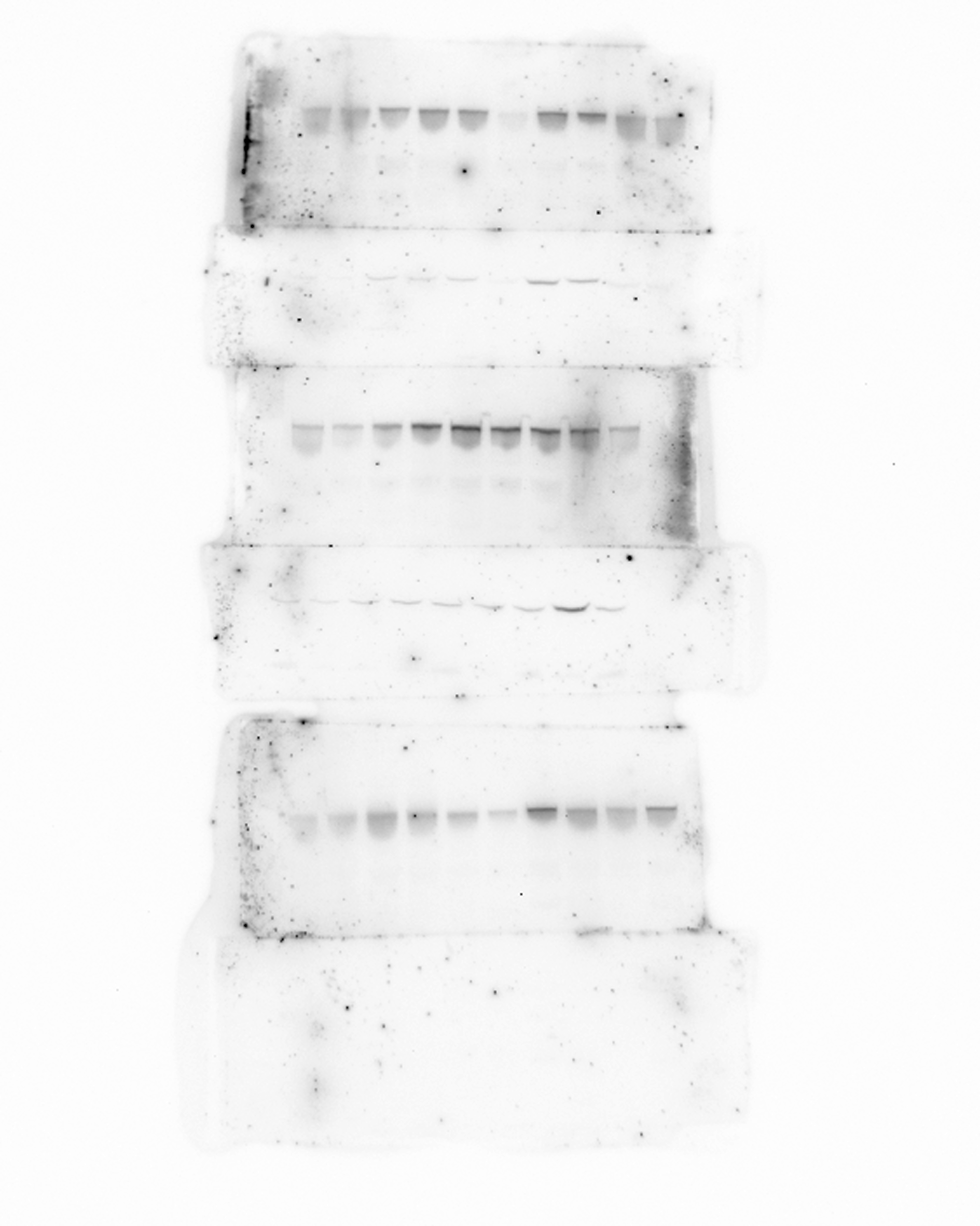

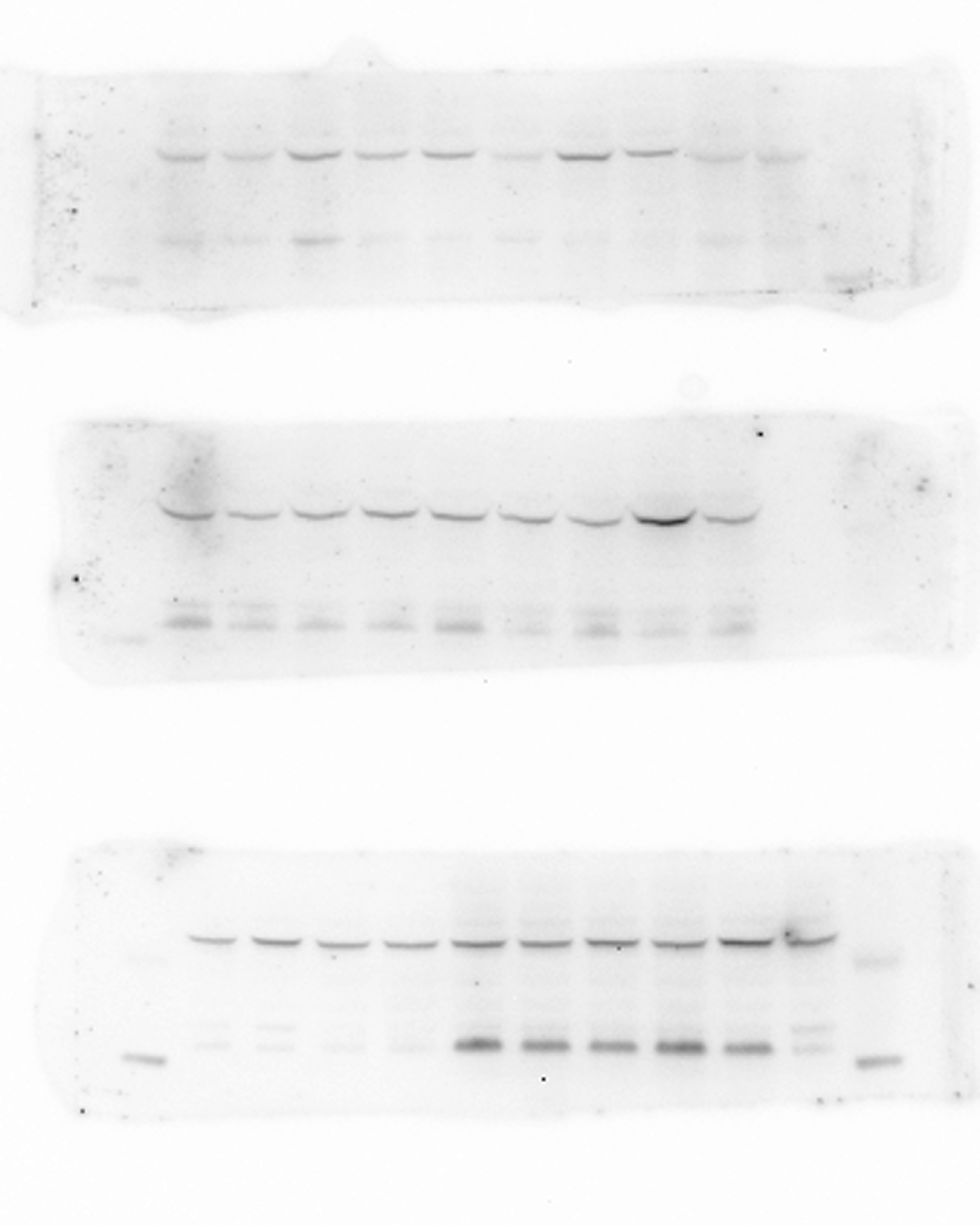


M

**1** 2 3

1 2 **3**

OVR

sepsis

OVR

sham

1 **2** 3

125 kD

OVR

sepsis+Lnd

45 kD

45 kD

M

sham

4 5

4 5

sepsis

OVR sepsis

4 5

4 5

4 5

OVR

sepsis+Lnd

OVR

sham

125 kD

*

*

*

*

*

*

*

*

*

*

*

*

*

*

*

*

*

*

x

x

x

**Supplemental Figure 3: Western blot assay for phosphorylated STAT3 and JAK2.**

Blot photos after incubation with antibodies specific for STA3 STAT3-P-Tyr705 (A), STAT3-P-Ser727 (B) and JAK2 (C). Before hybridization, membranes were cut according to the molecular weights of the tested proteins at the points indicated by the arrows. The samples marked with an asterisk are those used to illustrate Figure 3.Sepsis 3 sample was different from all other samples in the sepsis group and was excluded from the statistical analysis.

Lnd: Landiolol; OVR: Ovariectomized.
